# Supplementary material for: Modulation of sleep by trafficking of lipids through the Drosophila blood-brain barrier
Source: eLife. 2023 May 4;12:e86336. doi: 10.7554/eLife.86336 (PMC10205086; doi:10.7554/eLife.86336)
Supplement: Supplementary file 1. — All measured metabolites and their respective categories are listed for samples from Repo-GAL4>UAS-20xShibire, and both parental controls. Welch’s t-test was performed on scaled signal for each metabolite, comparing the conditions shown. Green highlighting marks a significant difference (p≤0.05) between the groups, where metabolite ratio is <1.00, while light green is not significant, but close to the threshold (0.05<p<0.10). Red highlighting marks a significant difference (p≤0.05) between groups where metabolite ratio is ≥1.00, and light red is not significant, but close to the threshold (0.05<p<0.10). [file elife-86336-supp1.docx]

| **Sub Pathway** | **Biochemical Name** | **Platform** | **Comp ID** | **KEGG** | **HMDB** | **PubChem** | **Gal4>UAS**  **GAL4 Ctrl** | **Gal4>UAS**  **UAS Ctrl** | **UAS Ctrl GAL4 Ctrl** |
| --- | --- | --- | --- | --- | --- | --- | --- | --- | --- |
| Glycine, Serine and Threonine Metabolism | glycine | LC/MS pos early | 58 | [C00037](http://www.genome.jp/dbget-bin/www_bget?cpd%2BC00037) | [HMDB00123](http://www.hmdb.ca/metabolites/HMDB00123) | 750 | **0.73** | 0.90 | **0.81** |
|  | N-acetylglycine | LC/MS polar | 27710 |  | [HMDB00532](http://www.hmdb.ca/metabolites/HMDB00532) | 10972 | **0.77** | 0.96 | **0.80** |
|  | sarcosine | LC/MS pos early | 1516 | [C00213](http://www.genome.jp/dbget-bin/www_bget?cpd%2BC00213) | [HMDB00271](http://www.hmdb.ca/metabolites/HMDB00271) | 1088 | **0.36** | 1.12 | **0.33** |
|  | betaine | LC/MS pos early | 3141 | [C00719](http://www.genome.jp/dbget-bin/www_bget?cpd%2BC00719) | [HMDB00043](http://www.hmdb.ca/metabolites/HMDB00043) | 247 | **0.56** | **0.56** | 1.01 |
|  | serine | LC/MS pos early | 1648 | [C00065](http://www.genome.jp/dbget-bin/www_bget?cpd%2BC00065) | [HMDB00187](http://www.hmdb.ca/metabolites/HMDB00187) | 5951 | 1.09 | 1.01 | 1.08 |
|  | N-acetylserine | LC/MS pos early | 37076 |  | [HMDB02931](http://www.hmdb.ca/metabolites/HMDB02931) | 65249 | 0.87 | 1.02 | **0.85** |
|  | 2-methylserine | LC/MS pos early | 53229 | [C02115](http://www.genome.jp/dbget-bin/www_bget?cpd%2BC02115) |  | 94309 | **0.75** | **0.50** | **1.49** |
|  | threonine | LC/MS pos early | 1284 | [C00188](http://www.genome.jp/dbget-bin/www_bget?cpd%2BC00188) | [HMDB00167](http://www.hmdb.ca/metabolites/HMDB00167) | 6288 | **0.72** | 0.94 | **0.77** |
|  | N-acetylthreonine | LC/MS polar | 33939 |  |  | 152204 | **0.56** | 0.90 | **0.63** |
|  | homoserine | LC/MS pos early | 18351 | [C00263](http://www.genome.jp/dbget-bin/www_bget?cpd%2BC00263) | [HMDB00719](http://www.hmdb.ca/metabolites/HMDB00719) | 12647 | 1.27 | 0.79 | **1.61** |
| Alanine and Aspartate Metabolism | alanine | LC/MS pos early | 1126 | [C00041](http://www.genome.jp/dbget-bin/www_bget?cpd%2BC00041) | [HMDB00161](http://www.hmdb.ca/metabolites/HMDB00161) | 5950 | 0.98 | 0.96 | 1.03 |
|  | N-acetylalanine | LC/MS polar | 1585 | [C02847](http://www.genome.jp/dbget-bin/www_bget?cpd%2BC02847) | [HMDB00766](http://www.hmdb.ca/metabolites/HMDB00766) | 88064 | **0.73** | **0.89** | **0.82** |
|  | N-methylalanine | LC/MS pos early | 37069 | [C02721](http://www.genome.jp/dbget-bin/www_bget?cpd%2BC02721) | [HMDB01906](http://www.hmdb.ca/metabolites/HMDB01906) | 5288725 | **0.59** | **0.63** | 0.93 |
|  | N-acetylaspartate (NAA) | LC/MS polar | 22185 | [C01042](http://www.genome.jp/dbget-bin/www_bget?cpd%2BC01042) | [HMDB00812](http://www.hmdb.ca/metabolites/HMDB00812) | 65065 | 1.46 | 1.12 | 1.30 |
|  | asparagine | LC/MS pos early | 512 | [C00152](http://www.genome.jp/dbget-bin/www_bget?cpd%2BC00152) | [HMDB00168](http://www.hmdb.ca/metabolites/HMDB00168) | 6267 | 1.11 | **0.81** | **1.37** |
|  | N-acetylasparagine | LC/MS pos early | 33942 |  | [HMDB06028](http://www.hmdb.ca/metabolites/HMDB06028) | 99715 | **0.81** | **0.89** | 0.91 |
| Glutamate Metabolism | glutamate | LC/MS pos early | 57 | [C00025](http://www.genome.jp/dbget-bin/www_bget?cpd%2BC00025) | [HMDB00148](http://www.hmdb.ca/metabolites/HMDB00148) | 611 | **1.41** | 1.06 | **1.33** |
|  | glutamine | LC/MS pos early | 53 | [C00064](http://www.genome.jp/dbget-bin/www_bget?cpd%2BC00064) | [HMDB00641](http://www.hmdb.ca/metabolites/HMDB00641) | 5961 | 1.32 | 0.87 | **1.51** |
|  | alpha-ketoglutaramate* | LC/MS polar | 62101 |  |  |  | **1.84** | 0.96 | **1.92** |
|  | N-acetylglutamate | LC/MS polar | 15720 | [C00624](http://www.genome.jp/dbget-bin/www_bget?cpd%2BC00624) | [HMDB01138](http://www.hmdb.ca/metabolites/HMDB01138) | 70914 | **1.27** | **0.61** | **2.08** |
|  | N-acetylglutamine | LC/MS pos early | 33943 | [C02716](http://www.genome.jp/dbget-bin/www_bget?cpd%2BC02716) | [HMDB06029](http://www.hmdb.ca/metabolites/HMDB06029) | 182230 | **0.68** | 0.92 | **0.74** |
|  | glutamate, gamma-methyl ester | LC/MS pos early | 33487 |  | [HMDB61715](http://www.hmdb.ca/metabolites/HMDB61715) | 68662 | 0.80 | 0.86 | 0.93 |
|  | pyroglutamine* | LC/MS pos early | 46225 |  |  | 134508 | **0.34** | 0.95 | **0.36** |
|  | gamma-aminobutyrate (GABA) | LC/MS pos early | 1416 | [C00334](http://www.genome.jp/dbget-bin/www_bget?cpd%2BC00334) | [HMDB00112](http://www.hmdb.ca/metabolites/HMDB00112) | 119 | 1.14 | 1.03 | 1.11 |
|  | carboxyethyl-GABA | LC/MS pos early | 40007 |  | [HMDB02201](http://www.hmdb.ca/metabolites/HMDB02201) | 2572 | 0.76 | 1.15 | **0.66** |
|  | N-methyl-GABA | LC/MS pos early | 39577 | [C15987](http://www.genome.jp/dbget-bin/www_bget?cpd%2BC15987) |  | 70703 | 0.82 | **0.64** | 1.29 |
|  | propionylglutamine | LC/MS pos early | 54909 |  |  |  | **0.65** | 1.05 | **0.62** |
| Histidine Metabolism | histidine | LC/MS neg | 59 | [C00135](http://www.genome.jp/dbget-bin/www_bget?cpd%2BC00135) | [HMDB00177](http://www.hmdb.ca/metabolites/HMDB00177) | 6274 | **0.84** | **0.81** | 1.04 |
|  | 1-methylhistidine | LC/MS pos early | 30460 | [C01152](http://www.genome.jp/dbget-bin/www_bget?cpd%2BC01152) | [HMDB00001](http://www.hmdb.ca/metabolites/HMDB00001) | 92105 | 1.11 | **0.73** | **1.51** |
|  | 3-methylhistidine | LC/MS pos early | 15677 | [C01152](http://www.genome.jp/dbget-bin/www_bget?cpd%2BC01152) | [HMDB00479](http://www.hmdb.ca/metabolites/HMDB00479) | 64969 | **1.91** | **0.26** | **7.23** |
|  | N-acetylhistidine | LC/MS pos early | 33946 | [C02997](http://www.genome.jp/dbget-bin/www_bget?cpd%2BC02997) | [HMDB32055](http://www.hmdb.ca/metabolites/HMDB32055) | 75619 | **0.82** | **0.81** | 1.00 |
|  | imidazole propionate | LC/MS pos early | 40730 |  | [HMDB02271](http://www.hmdb.ca/metabolites/HMDB02271) | 70630 | **0.46** | 0.77 | **0.60** |
|  | imidazole lactate | LC/MS pos early | 15716 | [C05568](http://www.genome.jp/dbget-bin/www_bget?cpd%2BC05568) | [HMDB02320](http://www.hmdb.ca/metabolites/HMDB02320) | 440129 | **0.57** | **0.75** | **0.75** |
|  | histamine | LC/MS pos early | 1574 | [C00388](http://www.genome.jp/dbget-bin/www_bget?cpd%2BC00388) | [HMDB00870](http://www.hmdb.ca/metabolites/HMDB00870) | 774 | 1.05 | 0.93 | 1.13 |
|  | 4-imidazoleacetate | LC/MS pos early | 32349 | [C02835](http://www.genome.jp/dbget-bin/www_bget?cpd%2BC02835) | [HMDB02024](http://www.hmdb.ca/metabolites/HMDB02024) | 96215 | **0.86** | 0.92 | 0.94 |
|  | N-acetylhistamine | LC/MS pos early | 48679 | [C05135](http://www.genome.jp/dbget-bin/www_bget?cpd%2BC05135) | [HMDB13253](http://www.hmdb.ca/metabolites/HMDB13253) | 69602 | 1.92 | 1.64 | 1.17 |
| Lysine Metabolism | lysine | LC/MS pos early | 1301 | [C00047](http://www.genome.jp/dbget-bin/www_bget?cpd%2BC00047) | [HMDB00182](http://www.hmdb.ca/metabolites/HMDB00182) | 5962 | 1.31 | 1.10 | 1.19 |
|  | N6,N6,N6-trimethyllysine | LC/MS pos early | 1498 | [C03793](http://www.genome.jp/dbget-bin/www_bget?cpd%2BC03793) | [HMDB01325](http://www.hmdb.ca/metabolites/HMDB01325) | 440120 | 0.96 | 1.96 | 0.49 |
|  | 5-(galactosylhydroxy)-L-lysine | LC/MS pos early | 43582 |  |  |  | 1.05 | 0.88 | 1.19 |
|  | saccharopine | LC/MS polar | 1495 | [C00449](http://www.genome.jp/dbget-bin/www_bget?cpd%2BC00449) | [HMDB00279](http://www.hmdb.ca/metabolites/HMDB00279) | 160556 | 1.53 | 1.37 | 1.11 |
|  | pipecolate | LC/MS pos early | 1444 | [C00408](http://www.genome.jp/dbget-bin/www_bget?cpd%2BC00408) | [HMDB00070](http://www.hmdb.ca/metabolites/HMDB00070) | 849 | 0.74 | **1.22** | **0.61** |
|  | N-trimethyl 5-aminovalerate | LC/MS pos early | 57687 |  |  |  | **3.87** | 1.87 | 2.07 |
| Phenylalanine Metabolism | phenylalanine | LC/MS pos early | 64 | [C00079](http://www.genome.jp/dbget-bin/www_bget?cpd%2BC00079) | [HMDB00159](http://www.hmdb.ca/metabolites/HMDB00159) | 6140 | **0.81** | **0.90** | **0.90** |
|  | N-acetylphenylalanine | LC/MS neg | 33950 | [C03519](http://www.genome.jp/dbget-bin/www_bget?cpd%2BC03519) | [HMDB00512](http://www.hmdb.ca/metabolites/HMDB00512) | 74839 | **0.58** | 1.37 | **0.42** |
| Tyrosine Metabolism | tyrosine | LC/MS pos early | 1299 | [C00082](http://www.genome.jp/dbget-bin/www_bget?cpd%2BC00082) | [HMDB00158](http://www.hmdb.ca/metabolites/HMDB00158) | 6057 | **2.61** | 1.29 | **2.03** |
|  | dihydoxyphenylalanine (L-DOPA) | LC/MS pos early | 1576 | [C00355](http://www.genome.jp/dbget-bin/www_bget?cpd%2BC00355) | [HMDB00181](http://www.hmdb.ca/metabolites/HMDB00181) | 6047 | **1.41** | 1.04 | **1.36** |
|  | N-formylphenylalanine | LC/MS neg | 48433 |  |  | 759256 | 1.50 | 1.31 | 1.15 |
| Tryptophan Metabolism | tryptophan | LC/MS pos early | 54 | [C00078](http://www.genome.jp/dbget-bin/www_bget?cpd%2BC00078) | [HMDB00929](http://www.hmdb.ca/metabolites/HMDB00929) | 6305 | 1.17 | 1.02 | 1.15 |
|  | kynurenine | LC/MS pos early | 15140 | [C00328](http://www.genome.jp/dbget-bin/www_bget?cpd%2BC00328) | [HMDB00684](http://www.hmdb.ca/metabolites/HMDB00684) | 161166 | 2.29 | 1.44 | 1.59 |
|  | kynurenate | LC/MS neg | 1417 | [C01717](http://www.genome.jp/dbget-bin/www_bget?cpd%2BC01717) | [HMDB00715](http://www.hmdb.ca/metabolites/HMDB00715) | 3845 | **3.21** | **2.14** | **1.50** |
|  | 3-hydroxykynurenine | LC/MS pos early | 22110 | [C02794](http://www.genome.jp/dbget-bin/www_bget?cpd%2BC02794) | [HMDB00732](http://www.hmdb.ca/metabolites/HMDB00732) | 89 | **4.59** | **1.17** | **3.94** |
|  | xanthurenate | LC/MS neg | 15679 | [C02470](http://www.genome.jp/dbget-bin/www_bget?cpd%2BC02470) | [HMDB00881](http://www.hmdb.ca/metabolites/HMDB00881) | 5699 | **2.05** | 1.07 | **1.91** |
|  | N-acetylserotonin | LC/MS neg | 1500 | [C00978](http://www.genome.jp/dbget-bin/www_bget?cpd%2BC00978) | [HMDB01238](http://www.hmdb.ca/metabolites/HMDB01238) | 903 | 0.81 | 0.99 | 0.82 |
| Leucine, Isoleucine and Valine Metabolism | leucine | LC/MS pos early | 60 |  |  | 5246661 | **0.64** | 0.95 | **0.68** |
|  | N-acetylleucine | LC/MS neg | 1587 | [C02710](http://www.genome.jp/dbget-bin/www_bget?cpd%2BC02710) | [HMDB11756](http://www.hmdb.ca/metabolites/HMDB11756) | 70912 | 0.61 | 1.14 | 0.53 |
|  | 4-methyl-2-oxopentanoate | LC/MS neg | 22116 | [C00233](http://www.genome.jp/dbget-bin/www_bget?cpd%2BC00233) | [HMDB00695](http://www.hmdb.ca/metabolites/HMDB00695) | 70 | 0.61 | 1.17 | 0.52 |
|  | beta-hydroxyisovalerate | LC/MS polar | 12129 |  | [HMDB00754](http://www.hmdb.ca/metabolites/HMDB00754) | 69362 | **0.81** | 1.05 | **0.78** |
|  | isoleucine | LC/MS pos early | 1125 | [C00407](http://www.genome.jp/dbget-bin/www_bget?cpd%2BC00407) | [HMDB00172](http://www.hmdb.ca/metabolites/HMDB00172) | 6306 | **0.71** | 0.94 | **0.76** |
|  | 3-methyl-2-oxovalerate | LC/MS neg | 15676 | [C00671](http://www.genome.jp/dbget-bin/www_bget?cpd%2BC00671) | [HMDB03736](http://www.hmdb.ca/metabolites/HMDB03736) | 47 | 0.75 | 1.16 | 0.65 |
|  | alpha-hydroxyisovalerate | LC/MS polar | 46537 |  | [HMDB00407](http://www.hmdb.ca/metabolites/HMDB00407) | 99823 | 0.81 | 0.96 | 0.84 |
|  | ethylmalonate | LC/MS polar | 15765 |  | [HMDB00622](http://www.hmdb.ca/metabolites/HMDB00622) | 11756 | 0.81 | 0.83 | 0.98 |
|  | methylsuccinate | LC/MS polar | 15745 |  | [HMDB01844](http://www.hmdb.ca/metabolites/HMDB01844) | 10349 | 0.96 | 0.82 | 1.18 |
|  | valine | LC/MS neg | 1649 | [C00183](http://www.genome.jp/dbget-bin/www_bget?cpd%2BC00183) | [HMDB00883](http://www.hmdb.ca/metabolites/HMDB00883) | 6287 | **0.62** | 0.87 | **0.72** |
|  | 3-methyl-2-oxobutyrate | LC/MS polar | 44526 | [C00141](http://www.genome.jp/dbget-bin/www_bget?cpd%2BC00141) | [HMDB00019](http://www.hmdb.ca/metabolites/HMDB00019) | 49 | 0.88 | 1.28 | 0.68 |
|  | 3-hydroxyisobutyrate | LC/MS polar | 1549 | [C06001](http://www.genome.jp/dbget-bin/www_bget?cpd%2BC06001) | [HMDB00336](http://www.hmdb.ca/metabolites/HMDB00336) | 87 | 0.72 | 1.09 | **0.66** |
| Methionine, Cysteine, SAM and Taurine Metabolism | methionine | LC/MS pos early | 1302 | [C00073](http://www.genome.jp/dbget-bin/www_bget?cpd%2BC00073) | [HMDB00696](http://www.hmdb.ca/metabolites/HMDB00696) | 6137 | **0.75** | 1.08 | **0.70** |
|  | N-acetylmethionine | LC/MS neg | 1589 | [C02712](http://www.genome.jp/dbget-bin/www_bget?cpd%2BC02712) | [HMDB11745](http://www.hmdb.ca/metabolites/HMDB11745) | 448580 | 0.54 | 0.96 | 0.57 |
|  | N-formylmethionine | LC/MS neg | 2829 | [C03145](http://www.genome.jp/dbget-bin/www_bget?cpd%2BC03145) | [HMDB01015](http://www.hmdb.ca/metabolites/HMDB01015) | 439750 | 0.76 | 1.53 | **0.50** |
|  | methionine sulfoxide | LC/MS pos early | 18374 | [C02989](http://www.genome.jp/dbget-bin/www_bget?cpd%2BC02989) | [HMDB02005](http://www.hmdb.ca/metabolites/HMDB02005) | 158980 | 0.57 | 0.75 | 0.77 |
|  | N-acetylmethionine sulfoxide | LC/MS pos early | 45428 |  |  | 193368 | 0.60 | 0.90 | 0.67 |
|  | S-adenosylhomocysteine (SAH) | LC/MS neg | 42382 | [C00021](http://www.genome.jp/dbget-bin/www_bget?cpd%2BC00021) | [HMDB00939](http://www.hmdb.ca/metabolites/HMDB00939) | 439155 | 1.14 | 1.22 | 0.93 |
|  | cystathionine | LC/MS pos early | 15705 | [C02291](http://www.genome.jp/dbget-bin/www_bget?cpd%2BC02291) | [HMDB00099](http://www.hmdb.ca/metabolites/HMDB00099) | 439258 | 0.97 | 1.14 | **0.85** |
|  | cysteine | LC/MS pos early | 1868 | [C00097](http://www.genome.jp/dbget-bin/www_bget?cpd%2BC00097) | [HMDB00574](http://www.hmdb.ca/metabolites/HMDB00574) | 5862 | **0.49** | **0.73** | **0.67** |
|  | N-acetylcysteine | LC/MS pos early | 1586 | [C06809](http://www.genome.jp/dbget-bin/www_bget?cpd%2BC06809) | [HMDB01890](http://www.hmdb.ca/metabolites/HMDB01890) | 12035 | 0.87 | 0.89 | 0.97 |
|  | S-methylcysteine sulfoxide | LC/MS pos early | 43378 |  | [HMDB29432](http://www.hmdb.ca/metabolites/HMDB29432) | 82142 | 1.12 | **0.71** | **1.58** |
|  | cystine | LC/MS neg | 56 | [C00491](http://www.genome.jp/dbget-bin/www_bget?cpd%2BC00491) | [HMDB00192](http://www.hmdb.ca/metabolites/HMDB00192) | 67678 | 0.63 | 0.64 | 1.00 |
|  | lanthionine | LC/MS pos early | 42002 |  |  | 98504 | 0.75 | 1.48 | 0.50 |
|  | cysteine sulfinic acid | LC/MS pos early | 37443 | [C00606](http://www.genome.jp/dbget-bin/www_bget?cpd%2BC00606) | [HMDB00996](http://www.hmdb.ca/metabolites/HMDB00996) | 109 | **1.84** | 0.87 | **2.12** |
|  | taurine | LC/MS neg | 2125 | [C00245](http://www.genome.jp/dbget-bin/www_bget?cpd%2BC00245) | [HMDB00251](http://www.hmdb.ca/metabolites/HMDB00251) | 1123 | 0.96 | **0.88** | 1.09 |
|  | N-acetyltaurine | LC/MS neg | 48187 |  |  | 159864 | 1.02 | 0.85 | **1.20** |
|  | cyano-alanine | LC/MS polar | 35660 | [C02512](http://www.genome.jp/dbget-bin/www_bget?cpd%2BC02512) |  | 13538 | 1.00 | **0.77** | **1.31** |

| **Sub Pathway** | **Biochemical Name** | **Platform** | **Comp ID** | **KEGG** | **HMDB** | **PubChem** | **Gal4>UAS**  **GAL4 Ctrl** | **Gal4>UAS**  **UAS Ctrl** | **UAS Ctrl GAL4 Ctrl** |
| --- | --- | --- | --- | --- | --- | --- | --- | --- | --- |
| Urea cycle; Arginine and Proline Metabolism | arginine | LC/MS pos early | 1638 | [C00062](http://www.genome.jp/dbget-bin/www_bget?cpd%2BC00062) | [HMDB00517](http://www.hmdb.ca/metabolites/HMDB00517) | 232 | 0.99 | 0.96 | 1.03 |
|  | argininosuccinate | LC/MS pos early | 15497 | [C03406](http://www.genome.jp/dbget-bin/www_bget?cpd%2BC03406) | [HMDB00052](http://www.hmdb.ca/metabolites/HMDB00052) | 828 | 1.11 | 1.23 | 0.90 |
|  | ornithine | LC/MS pos early | 1493 | [C00077](http://www.genome.jp/dbget-bin/www_bget?cpd%2BC00077) | [HMDB03374](http://www.hmdb.ca/metabolites/HMDB03374) | 6262 | 1.38 | **1.38** | 1.00 |
|  | 2-oxoarginine* | LC/MS pos early | 55072 | [C03771](http://www.genome.jp/dbget-bin/www_bget?cpd%2BC03771) | [HMDB04225](http://www.hmdb.ca/metabolites/HMDB04225) | 558 | 0.73 | 1.00 | 0.74 |
|  | citrulline | LC/MS pos early | 2132 | [C00327](http://www.genome.jp/dbget-bin/www_bget?cpd%2BC00327) | [HMDB00904](http://www.hmdb.ca/metabolites/HMDB00904) | 9750 | **0.73** | 0.97 | **0.75** |
|  | proline | LC/MS pos early | 1898 | [C00148](http://www.genome.jp/dbget-bin/www_bget?cpd%2BC00148) | [HMDB00162](http://www.hmdb.ca/metabolites/HMDB00162) | 145742 | **1.31** | 1.02 | **1.28** |
|  | dimethylarginine (SDMA + ADMA) | LC/MS pos early | 36808 | [C03626](http://www.genome.jp/dbget-bin/www_bget?cpd%2BC03626) | [HMDB01539](http://www.hmdb.ca/metabolites/HMDB01539) | 123831 | 1.38 | 1.09 | 1.27 |
|  | N-acetylarginine | LC/MS pos early | 33953 | [C02562](http://www.genome.jp/dbget-bin/www_bget?cpd%2BC02562) | [HMDB04620](http://www.hmdb.ca/metabolites/HMDB04620) | 67427 | 1.72 | 1.72 | 1.00 |
|  | N-delta-acetylornithine | LC/MS pos early | 43249 |  |  | 9920500 | **1.35** | 1.09 | 1.24 |
|  | N-alpha-acetylornithine | LC/MS pos early | 32984 | [C00437](http://www.genome.jp/dbget-bin/www_bget?cpd%2BC00437) | [HMDB03357](http://www.hmdb.ca/metabolites/HMDB03357) | 439232 | **1.54** | **1.48** | 1.04 |
|  | trans-4-hydroxyproline | LC/MS pos early | 32306 | [C01157](http://www.genome.jp/dbget-bin/www_bget?cpd%2BC01157) | [HMDB00725](http://www.hmdb.ca/metabolites/HMDB00725) | 5810 | 0.77 | **0.62** | 1.23 |
|  | argininate* | LC/MS pos early | 57461 |  | [HMDB03148](http://www.hmdb.ca/metabolites/HMDB03148) | 160437 | 0.75 | 1.15 | **0.65** |
| Polyamine Metabolism | putrescine | LC/MS pos early | 1408 | [C00134](http://www.genome.jp/dbget-bin/www_bget?cpd%2BC00134) | [HMDB01414](http://www.hmdb.ca/metabolites/HMDB01414) | 1045 | **5.09** | 0.83 | **6.14** |
|  | N-acetyl-isoputreanine* | LC/MS pos early | 62309 |  |  |  | 1.00 | 1.00 | 1.00 |
|  | spermidine | LC/MS pos early | 485 | [C00315](http://www.genome.jp/dbget-bin/www_bget?cpd%2BC00315) | [HMDB01257](http://www.hmdb.ca/metabolites/HMDB01257) | 1102 | **1.23** | 0.99 | **1.25** |
|  | 5-methylthioadenosine (MTA) | LC/MS pos early | 1419 | [C00170](http://www.genome.jp/dbget-bin/www_bget?cpd%2BC00170) | [HMDB01173](http://www.hmdb.ca/metabolites/HMDB01173) | 439176 | **1.48** | **1.17** | 1.26 |
|  | 4-acetamidobutanoate | LC/MS pos early | 1558 | [C02946](http://www.genome.jp/dbget-bin/www_bget?cpd%2BC02946) | [HMDB03681](http://www.hmdb.ca/metabolites/HMDB03681) | 18189 | 0.89 | 0.86 | 1.03 |
|  | (N(1) + N(8))-acetylspermidine | LC/MS pos early | 57814 |  |  |  | 0.79 | 0.91 | 0.86 |
| Guanidino and Acetamido Metabolism | 4-guanidinobutanoate | LC/MS pos early | 15681 | [C01035](http://www.genome.jp/dbget-bin/www_bget?cpd%2BC01035) | [HMDB03464](http://www.hmdb.ca/metabolites/HMDB03464) | 500 | 1.00 | 1.14 | 0.87 |
| Glutathione Metabolism | glutathione, reduced (GSH) | LC/MS pos early | 2127 | [C00051](http://www.genome.jp/dbget-bin/www_bget?cpd%2BC00051) | [HMDB00125](http://www.hmdb.ca/metabolites/HMDB00125) | 124886 | **4.09** | 0.83 | **4.94** |
|  | cysteine-glutathione disulfide | LC/MS pos early | 35159 |  | [HMDB00656](http://www.hmdb.ca/metabolites/HMDB00656) | 4247235 | 1.72 | 0.82 | **2.10** |
|  | cysteinylglycine | LC/MS pos early | 35637 | [C01419](http://www.genome.jp/dbget-bin/www_bget?cpd%2BC01419) | [HMDB00078](http://www.hmdb.ca/metabolites/HMDB00078) | 439498 | **0.36** | 0.49 | 0.73 |
|  | cysteinylglycine disulfide* | LC/MS pos early | 62103 |  | [HMDB00709](http://www.hmdb.ca/metabolites/HMDB00709) |  | **1.89** | 0.88 | **2.15** |
|  | 5-oxoproline | LC/MS neg | 1494 | [C01879](http://www.genome.jp/dbget-bin/www_bget?cpd%2BC01879) | [HMDB00267](http://www.hmdb.ca/metabolites/HMDB00267) | 7405 | **0.64** | **0.79** | **0.80** |
| Gamma-glutamyl Amino Acid | gamma-glutamylalanine | LC/MS pos early | 37063 |  | [HMDB29142](http://www.hmdb.ca/metabolites/HMDB29142) | 440103 | 0.88 | 0.91 | 0.97 |
|  | gamma-glutamylcysteine | LC/MS pos early | 1778 | [C00669](http://www.genome.jp/dbget-bin/www_bget?cpd%2BC00669) | [HMDB01049](http://www.hmdb.ca/metabolites/HMDB01049) | 842 | **0.25** | 0.58 | **0.43** |
|  | gamma-glutamylglutamate | LC/MS pos early | 36738 | [C05282](http://www.genome.jp/dbget-bin/www_bget?cpd%2BC05282) | [HMDB11737](http://www.hmdb.ca/metabolites/HMDB11737) | 92865 | 0.90 | **0.83** | 1.08 |
|  | gamma-glutamylglutamine | LC/MS pos early | 2730 | [C05283](http://www.genome.jp/dbget-bin/www_bget?cpd%2BC05283) | [HMDB11738](http://www.hmdb.ca/metabolites/HMDB11738) | 150914 | **1.66** | 1.13 | **1.47** |
|  | gamma-glutamylglycine | LC/MS pos early | 33949 |  | [HMDB11667](http://www.hmdb.ca/metabolites/HMDB11667) | 165527 | **0.37** | **0.63** | **0.59** |
|  | gamma-glutamylhistidine | LC/MS pos early | 18245 |  |  | 7017195 | **0.55** | **0.75** | **0.73** |
|  | gamma-glutamylisoleucine* | LC/MS neg | 34457 |  | [HMDB11170](http://www.hmdb.ca/metabolites/HMDB11170) | 14253342 | **0.40** | 1.01 | **0.39** |
|  | gamma-glutamylleucine | LC/MS neg | 18369 |  | [HMDB11171](http://www.hmdb.ca/metabolites/HMDB11171) | 151023 | **0.45** | 0.87 | **0.52** |
|  | gamma-glutamyl-alpha-lysine | LC/MS pos early | 55015 |  |  | 65254 | **1.29** | **1.32** | 0.98 |
|  | gamma-glutamylmethionine | LC/MS pos early | 44872 |  | [HMDB29155](http://www.hmdb.ca/metabolites/HMDB29155) | 7009567 | **0.43** | **1.40** | **0.31** |
|  | gamma-glutamylthreonine | LC/MS pos early | 33364 |  | [HMDB29159](http://www.hmdb.ca/metabolites/HMDB29159) | 76078708 | **0.61** | 0.94 | **0.65** |
|  | gamma-glutamylvaline | LC/MS pos early | 43829 |  | [HMDB11172](http://www.hmdb.ca/metabolites/HMDB11172) | 7015683 | **0.48** | 0.70 | **0.69** |
| Dipeptide | alanylalanine | LC/MS pos early | 15129 |  | [HMDB28680](http://www.hmdb.ca/metabolites/HMDB28680) | 5484352 | 1.13 | 0.91 | 1.24 |
|  | alanylglutamate | LC/MS pos early | 37064 |  |  | 656476 | 0.84 | **0.84** | 1.00 |
|  | alanylproline | LC/MS pos early | 37083 |  | [HMDB28695](http://www.hmdb.ca/metabolites/HMDB28695) | 418040 | 0.83 | 1.21 | 0.69 |
|  | alanylthreonine | LC/MS pos early | 37085 |  | [HMDB28697](http://www.hmdb.ca/metabolites/HMDB28697) | 426318 | 1.05 | 0.98 | 1.06 |
|  | alpha-glutamylalanine | LC/MS pos early | 41369 |  | [HMDB03764](http://www.hmdb.ca/metabolites/HMDB03764) | 100098 | 0.90 | 0.93 | 0.97 |
|  | alpha-glutamylglutamate | LC/MS pos early | 22166 | [C01425](http://www.genome.jp/dbget-bin/www_bget?cpd%2BC01425) | [HMDB28818](http://www.hmdb.ca/metabolites/HMDB28818) | 439500 | 0.87 | 1.07 | **0.81** |
|  | asparaginylalanine | LC/MS pos early | 54731 |  | [HMDB28724](http://www.hmdb.ca/metabolites/HMDB28724) |  | 1.12 | 0.98 | 1.14 |
|  | glutaminylglutamate | LC/MS pos early | 43025 |  |  |  | **0.71** | 0.94 | **0.76** |
|  | serylthreonine | LC/MS pos early | 54732 |  | [HMDB29049](http://www.hmdb.ca/metabolites/HMDB29049) |  | **0.69** | 0.92 | **0.74** |
|  | glycylglycine | LC/MS pos early | 21029 | [C02037](http://www.genome.jp/dbget-bin/www_bget?cpd%2BC02037) | [HMDB11733](http://www.hmdb.ca/metabolites/HMDB11733) | 11163 | 0.86 | 1.05 | 0.82 |
|  | glycylisoleucine | LC/MS pos early | 36659 |  | [HMDB28844](http://www.hmdb.ca/metabolites/HMDB28844) | 88079 | 1.04 | 0.88 | 1.18 |
|  | glycylleucine | LC/MS pos early | 34398 | [C02155](http://www.genome.jp/dbget-bin/www_bget?cpd%2BC02155) | [HMDB00759](http://www.hmdb.ca/metabolites/HMDB00759) | 92843 | 0.89 | 0.83 | 1.07 |
|  | glycylphenylalanine | LC/MS neg | 33954 |  | [HMDB28848](http://www.hmdb.ca/metabolites/HMDB28848) | 92953 | 1.24 | 1.19 | 1.04 |
|  | glycylproline | LC/MS pos early | 22171 |  | [HMDB00721](http://www.hmdb.ca/metabolites/HMDB00721) | 3013625 | **0.69** | 0.75 | 0.92 |
|  | glycylvaline | LC/MS pos early | 18357 |  | [HMDB28854](http://www.hmdb.ca/metabolites/HMDB28854) | 97417 | **0.67** | 0.83 | 0.81 |
|  | isoleucylalanine | LC/MS pos early | 40046 |  | [HMDB28900](http://www.hmdb.ca/metabolites/HMDB28900) | 5246009 | 1.72 | 0.99 | **1.73** |
|  | isoleucylglutamate | LC/MS pos early | 40057 |  |  |  | 0.96 | 1.23 | 0.79 |
|  | isoleucylglutamine | LC/MS pos early | 40019 |  |  | 7020102 | **1.60** | 1.05 | **1.52** |
|  | isoleucylglycine | LC/MS neg | 40008 |  | [HMDB28907](http://www.hmdb.ca/metabolites/HMDB28907) | 342532 | 1.15 | 1.09 | 1.05 |
|  | isoleucylthreonine | LC/MS pos early | 42968 |  | [HMDB28917](http://www.hmdb.ca/metabolites/HMDB28917) | 16122515 | **1.46** | 0.95 | **1.53** |
|  | leucylalanine | LC/MS pos early | 40010 |  | [HMDB28922](http://www.hmdb.ca/metabolites/HMDB28922) | 259321 | **4.22** | 1.01 | **4.19** |
|  | leucylglycine | LC/MS pos early | 40045 |  | [HMDB28929](http://www.hmdb.ca/metabolites/HMDB28929) | 79070 | **1.67** | 0.96 | **1.73** |
|  | leucylleucine | LC/MS pos early | 36756 | [C11332](http://www.genome.jp/dbget-bin/www_bget?cpd%2BC11332) | [HMDB28933](http://www.hmdb.ca/metabolites/HMDB28933) | 76807 | **5.53** | 0.92 | **6.02** |
|  | leucylproline | LC/MS pos early | 35663 |  | [HMDB11175](http://www.hmdb.ca/metabolites/HMDB11175) | 80817 | **0.52** | 0.94 | **0.56** |
|  | leucylthreonine | LC/MS pos early | 42969 |  | [HMDB28939](http://www.hmdb.ca/metabolites/HMDB28939) | 10353878 | **2.16** | 0.91 | **2.37** |
|  | phenylalanylglutamate | LC/MS neg | 41432 |  |  | 4422358 | 0.87 | 0.91 | 0.96 |
|  | prolylalanine | LC/MS pos early | 40705 |  | [HMDB29010](http://www.hmdb.ca/metabolites/HMDB29010) | 418041 | 0.87 | 0.96 | 0.90 |
|  | prolylglutamine | LC/MS pos early | 40659 |  |  |  | 1.11 | 0.92 | 1.21 |
|  | prolylglycine | LC/MS pos early | 40703 |  | [HMDB11178](http://www.hmdb.ca/metabolites/HMDB11178) | 6426709 | 0.86 | 1.01 | 0.85 |
|  | prolylleucine | LC/MS pos early | 31914 |  |  | 3527720 | 0.94 | 0.92 | 1.03 |
|  | prolylproline | LC/MS pos early | 40731 |  | [HMDB11180](http://www.hmdb.ca/metabolites/HMDB11180) | 11622593 | 0.71 | 0.89 | 0.80 |
|  | prolylthreonine | LC/MS pos early | 44551 |  | [HMDB29027](http://www.hmdb.ca/metabolites/HMDB29027) |  | 0.74 | 1.12 | 0.67 |
|  | prolylvaline | LC/MS pos early | 40720 |  |  | 152307 | **0.72** | 0.81 | 0.89 |
|  | serylalanine | LC/MS pos early | 42049 |  | [HMDB29032](http://www.hmdb.ca/metabolites/HMDB29032) | 17958834 | 1.07 | **0.85** | 1.26 |
|  | serylleucine | LC/MS pos early | 40066 |  | [HMDB29043](http://www.hmdb.ca/metabolites/HMDB29043) | 7015695 | 1.15 | 1.11 | 1.04 |
|  | serylproline | LC/MS pos early | 42055 |  | [HMDB29047](http://www.hmdb.ca/metabolites/HMDB29047) | 4369021 | 1.04 | 0.90 | 1.16 |
|  | serylserine | LC/MS pos early | 42053 |  |  | 138784 | 0.94 | **0.83** | 1.13 |
|  | serylvaline | LC/MS pos early | 42058 |  | [HMDB29052](http://www.hmdb.ca/metabolites/HMDB29052) | 7020159 | **1.45** | 0.95 | **1.54** |
|  | valylaspartate | LC/MS pos early | 40650 |  | [HMDB29123](http://www.hmdb.ca/metabolites/HMDB29123) | 9964657 | 0.91 | 0.92 | 0.99 |
|  | valylglycine | LC/MS neg | 40475 |  | [HMDB29127](http://www.hmdb.ca/metabolites/HMDB29127) | 136487 | 1.07 | 1.01 | 1.06 |
|  | valylproline | LC/MS pos early | 40485 |  | [HMDB29135](http://www.hmdb.ca/metabolites/HMDB29135) | 5003412 | **0.70** | 0.95 | **0.74** |
|  | isoleucylleucine/leucylisoleucine | LC/MS pos early | 52322 |  |  |  | **2.09** | 0.79 | **2.65** |
|  | alpha-glutamylproline* | LC/MS pos early | 57731 |  |  |  | 1.00 | 1.00 | 1.00 |
| Modified Peptides | pyroglutamylleucine* | LC/MS neg | 62096 |  |  |  | 0.94 | 1.26 | 0.75 |

| **Sub Pathway** | **Biochemical Name** | **Platform** | **Comp ID** | **KEGG** | **HMDB** | **PubChem** | **Gal4>UAS**  **GAL4 Ctrl** | **Gal4>UAS**  **UAS Ctrl** | **UAS Ctrl GAL4 Ctrl** |
| --- | --- | --- | --- | --- | --- | --- | --- | --- | --- |
| Glycolysis, Gluconeogenesis, and Pyruvate Metabolism | glucose | LC/MS polar | 48152 | [C00031](http://www.genome.jp/dbget-bin/www_bget?cpd%2BC00031) | [HMDB00122](http://www.hmdb.ca/metabolites/HMDB00122) | 79025 | 1.03 | 1.01 | 1.01 |
|  | fructose 1,6-diphosphate/glucose 1,6-diphosphate/myo-inositol diphosphates | LC/MS neg | 46896 | [C00354](http://www.genome.jp/dbget-bin/www_bget?cpd%2BC00354) |  |  | 1.62 | 0.84 | 1.93 |
|  | dihydroxyacetone phosphate (DHAP) | LC/MS neg | 15522 | [C00111](http://www.genome.jp/dbget-bin/www_bget?cpd%2BC00111) | [HMDB01473](http://www.hmdb.ca/metabolites/HMDB01473) | 668 | 1.48 | **1.68** | 0.88 |
|  | 3-phosphoglycerate | LC/MS neg | 1414 | [C00597](http://www.genome.jp/dbget-bin/www_bget?cpd%2BC00597) | [HMDB00807](http://www.hmdb.ca/metabolites/HMDB00807) | 724 | 0.86 | 0.81 | 1.06 |
|  | phosphoenolpyruvate (PEP) | LC/MS neg | 597 | [C00074](http://www.genome.jp/dbget-bin/www_bget?cpd%2BC00074) | [HMDB00263](http://www.hmdb.ca/metabolites/HMDB00263) | 1005 | 0.77 | 0.73 | 1.06 |
|  | pyruvate | LC/MS polar | 48990 | [C00022](http://www.genome.jp/dbget-bin/www_bget?cpd%2BC00022) | [HMDB00243](http://www.hmdb.ca/metabolites/HMDB00243) | 1060 | 1.01 | **0.86** | **1.17** |
|  | lactate | LC/MS polar | 527 | [C00186](http://www.genome.jp/dbget-bin/www_bget?cpd%2BC00186) | [HMDB00190](http://www.hmdb.ca/metabolites/HMDB00190) | 612 | **0.63** | **0.85** | **0.74** |
|  | glycerate | LC/MS polar | 1572 | [C00258](http://www.genome.jp/dbget-bin/www_bget?cpd%2BC00258) | [HMDB00139](http://www.hmdb.ca/metabolites/HMDB00139) | 752 | 1.04 | 1.12 | 0.93 |
| Pentose Phosphate Pathway | 6-phosphogluconate | LC/MS neg | 15442 | [C00345](http://www.genome.jp/dbget-bin/www_bget?cpd%2BC00345) | [HMDB01316](http://www.hmdb.ca/metabolites/HMDB01316) | 91493 | **0.60** | 0.71 | 0.86 |
|  | sedoheptulose-7-phosphate | LC/MS neg | 35649 | [C05382](http://www.genome.jp/dbget-bin/www_bget?cpd%2BC05382) | [HMDB01068](http://www.hmdb.ca/metabolites/HMDB01068) | 616 | 0.87 | **0.77** | 1.13 |
| Pentose Metabolism | ribose | LC/MS polar | 1471 | [C00121](http://www.genome.jp/dbget-bin/www_bget?cpd%2BC00121) | [HMDB00283](http://www.hmdb.ca/metabolites/HMDB00283) | 5779 | 0.94 | 1.01 | 0.94 |
|  | ribitol | LC/MS polar | 15772 | [C00474](http://www.genome.jp/dbget-bin/www_bget?cpd%2BC00474) | [HMDB00508](http://www.hmdb.ca/metabolites/HMDB00508) | 6912 | **0.01** | 0.87 | **0.02** |
|  | ribonate | LC/MS polar | 27731 | [C01685](http://www.genome.jp/dbget-bin/www_bget?cpd%2BC01685) | [HMDB00867](http://www.hmdb.ca/metabolites/HMDB00867) | 5460677 | 0.78 | 0.91 | 0.86 |
|  | arabitol/xylitol | LC/MS polar | 48885 | [C01904](http://www.genome.jp/dbget-bin/www_bget?cpd%2BC01904) |  | 6912 | 1.10 | 0.96 | 1.15 |
|  | ribulose/xylulose | LC/MS polar | 54671 |  |  | 5289590 | **0.74** | 0.94 | **0.79** |
|  | arabonate/xylonate | LC/MS polar | 48255 |  |  |  | 0.93 | 0.93 | 1.00 |
|  | sedoheptulose | LC/MS polar | 53237 |  | [HMDB03219](http://www.hmdb.ca/metabolites/HMDB03219) | 5459879 | 0.84 | 0.75 | 1.13 |
|  | ribulonate/xylulonate* | LC/MS polar | 61858 |  |  |  | 1.01 | 1.00 | 1.01 |
| Glycogen Metabolism | maltotetraose | LC/MS polar | 15910 | [C02052](http://www.genome.jp/dbget-bin/www_bget?cpd%2BC02052) | [HMDB01296](http://www.hmdb.ca/metabolites/HMDB01296) | 446495 | **0.68** | **0.74** | 0.91 |
|  | maltotriose | LC/MS polar | 44688 | [C01835](http://www.genome.jp/dbget-bin/www_bget?cpd%2BC01835) | [HMDB01262](http://www.hmdb.ca/metabolites/HMDB01262) | 439586 | 1.04 | **0.83** | **1.25** |
|  | maltose | LC/MS polar | 15586 | [C00208](http://www.genome.jp/dbget-bin/www_bget?cpd%2BC00208) | [HMDB00163](http://www.hmdb.ca/metabolites/HMDB00163) | 10991489 | **1.26** | **0.90** | **1.40** |
|  | isomaltose | LC/MS polar | 39777 | [C00252](http://www.genome.jp/dbget-bin/www_bget?cpd%2BC00252) | [HMDB02923](http://www.hmdb.ca/metabolites/HMDB02923) | 439193 | 1.02 | 1.13 | 0.91 |
| Fructose, Mannose and Galactose Metabolism | fructose | LC/MS polar | 48195 | [C00095](http://www.genome.jp/dbget-bin/www_bget?cpd%2BC00095) | [HMDB00660](http://www.hmdb.ca/metabolites/HMDB00660) | 5984 | 0.85 | 0.90 | 0.94 |
|  | mannitol/sorbitol | LC/MS polar | 46142 | [C00794](http://www.genome.jp/dbget-bin/www_bget?cpd%2BC00794) | [HMDB00247](http://www.hmdb.ca/metabolites/HMDB00247) | 5780 | **0.66** | 0.93 | **0.71** |
|  | mannose | LC/MS polar | 48153 | [C00159](http://www.genome.jp/dbget-bin/www_bget?cpd%2BC00159) | [HMDB00169](http://www.hmdb.ca/metabolites/HMDB00169) | 18950 | **0.71** | **0.62** | 1.15 |
|  | galactitol (dulcitol) | LC/MS polar | 1117 | [C01697](http://www.genome.jp/dbget-bin/www_bget?cpd%2BC01697) | [HMDB00107](http://www.hmdb.ca/metabolites/HMDB00107) | 11850 | 0.81 | 0.64 | 1.27 |
|  | galactonate | LC/MS polar | 27719 | [C00880](http://www.genome.jp/dbget-bin/www_bget?cpd%2BC00880) | [HMDB00565](http://www.hmdb.ca/metabolites/HMDB00565) | 128869 | 0.66 | 0.64 | 1.03 |
| Nucleotide Sugar | UDP-N-acetylglucosamine/galactosamine | LC/MS neg | 46148 |  |  |  | **0.81** | **0.81** | 1.00 |
| Aminosugar Metabolism | glucuronate | LC/MS polar | 15443 | [C00191](http://www.genome.jp/dbget-bin/www_bget?cpd%2BC00191) | [HMDB00127](http://www.hmdb.ca/metabolites/HMDB00127) | 444791 | **0.75** | 0.95 | 0.79 |
|  | N-acetylglucosamine 6-phosphate | LC/MS polar | 15107 | [C00357](http://www.genome.jp/dbget-bin/www_bget?cpd%2BC00357) | [HMDB02817](http://www.hmdb.ca/metabolites/HMDB02817) | 439219 | **2.39** | 0.90 | **2.67** |
|  | N-acetylglucosaminylasparagine | LC/MS pos early | 48149 | [C04540](http://www.genome.jp/dbget-bin/www_bget?cpd%2BC04540) | [HMDB00489](http://www.hmdb.ca/metabolites/HMDB00489) | 123826 | 1.26 | 0.93 | **1.36** |
|  | erythronate* | LC/MS polar | 42420 |  | [HMDB00613](http://www.hmdb.ca/metabolites/HMDB00613) | 2781043 | 0.90 | 0.98 | 0.91 |
|  | N-acetylglucosamine/N-acetylgalactosamine | LC/MS pos early | 46539 |  | [HMDB00215](http://www.hmdb.ca/metabolites/HMDB00215) | 24139 | 0.87 | 0.93 | 0.94 |
| Advanced Glycation End-product | N6-carboxymethyllysine | LC/MS pos early | 36713 |  |  | 123800 | 1.06 | 0.97 | 1.09 |
| TCA Cycle | citrate | LC/MS neg | 1564 | [C00158](http://www.genome.jp/dbget-bin/www_bget?cpd%2BC00158) | [HMDB00094](http://www.hmdb.ca/metabolites/HMDB00094) | 311 | 0.85 | 1.18 | **0.72** |
|  | aconitate [cis or trans] | LC/MS neg | 46173 |  |  |  | 0.95 | 0.92 | 1.03 |
|  | isocitric lactone | LC/MS polar | 54724 |  |  | 98259 | 1.59 | **1.85** | 0.86 |
|  | alpha-ketoglutarate | LC/MS polar | 528 | [C00026](http://www.genome.jp/dbget-bin/www_bget?cpd%2BC00026) | [HMDB00208](http://www.hmdb.ca/metabolites/HMDB00208) | 51 | **1.35** | 1.07 | **1.26** |
|  | succinate | LC/MS polar | 1437 | [C00042](http://www.genome.jp/dbget-bin/www_bget?cpd%2BC00042) | [HMDB00254](http://www.hmdb.ca/metabolites/HMDB00254) | 1110 | **1.24** | **0.87** | **1.41** |
|  | fumarate | LC/MS polar | 1643 | [C00122](http://www.genome.jp/dbget-bin/www_bget?cpd%2BC00122) | [HMDB00134](http://www.hmdb.ca/metabolites/HMDB00134) | 444972 | 0.71 | 1.04 | **0.69** |
|  | malate | LC/MS neg | 1303 | [C00149](http://www.genome.jp/dbget-bin/www_bget?cpd%2BC00149) | [HMDB00156](http://www.hmdb.ca/metabolites/HMDB00156) | 525 | 0.76 | 0.97 | **0.78** |
|  | itaconate | LC/MS polar | 18373 | [C00490](http://www.genome.jp/dbget-bin/www_bget?cpd%2BC00490) | [HMDB02092](http://www.hmdb.ca/metabolites/HMDB02092) | 811 | 0.92 | **0.59** | **1.58** |
|  | tricarballylate | LC/MS polar | 15729 | [C19806](http://www.genome.jp/dbget-bin/www_bget?cpd%2BC19806) | [HMDB31193](http://www.hmdb.ca/metabolites/HMDB31193) | 14925 | 0.90 | 0.92 | 0.98 |
|  | 2-methylcitrate | LC/MS neg | 37483 | [C02225](http://www.genome.jp/dbget-bin/www_bget?cpd%2BC02225) | [HMDB00379](http://www.hmdb.ca/metabolites/HMDB00379) | 439681 | 1.09 | 1.03 | 1.06 |
|  | mesaconate (methylfumarate) | LC/MS polar | 18493 | [C01732](http://www.genome.jp/dbget-bin/www_bget?cpd%2BC01732) | [HMDB00749](http://www.hmdb.ca/metabolites/HMDB00749) | 638129 | 0.91 | **0.62** | **1.46** |
| Oxidative Phosphorylation | acetylphosphate | LC/MS polar | 15488 | [C00227](http://www.genome.jp/dbget-bin/www_bget?cpd%2BC00227) | [HMDB01494](http://www.hmdb.ca/metabolites/HMDB01494) | 186 | 1.13 | 0.71 | 1.60 |
|  | phosphate | LC/MS pos early | 42109 | [C00009](http://www.genome.jp/dbget-bin/www_bget?cpd%2BC00009) | [HMDB01429](http://www.hmdb.ca/metabolites/HMDB01429) | 1061 | 1.00 | 0.99 | 1.01 |
| Fatty Acid Synthesis | malonate | LC/MS polar | 15872 | [C00383](http://www.genome.jp/dbget-bin/www_bget?cpd%2BC00383) | [HMDB00691](http://www.hmdb.ca/metabolites/HMDB00691) | 867 | 0.85 | 0.86 | 1.00 |
| Medium Chain Fatty Acid | caprate (10:0) | LC/MS neg | 1642 | [C01571](http://www.genome.jp/dbget-bin/www_bget?cpd%2BC01571) | [HMDB00511](http://www.hmdb.ca/metabolites/HMDB00511) | 2969 | 1.45 | 1.04 | 1.40 |
|  | laurate (12:0) | LC/MS neg | 1645 | [C02679](http://www.genome.jp/dbget-bin/www_bget?cpd%2BC02679) | [HMDB00638](http://www.hmdb.ca/metabolites/HMDB00638) | 3893 | 0.79 | 1.08 | **0.73** |
|  | 5-dodecenoate (12:1n7) | LC/MS neg | 33968 |  | [HMDB00529](http://www.hmdb.ca/metabolites/HMDB00529) | 5312378 | 1.17 | 1.14 | 1.03 |
| Long Chain Fatty Acid | myristate (14:0) | LC/MS neg | 1365 | [C06424](http://www.genome.jp/dbget-bin/www_bget?cpd%2BC06424) | [HMDB00806](http://www.hmdb.ca/metabolites/HMDB00806) | 11005 | 1.18 | 1.04 | 1.14 |
|  | myristoleate (14:1n5) | LC/MS neg | 32418 | [C08322](http://www.genome.jp/dbget-bin/www_bget?cpd%2BC08322) | [HMDB02000](http://www.hmdb.ca/metabolites/HMDB02000) | 5281119 | 0.99 | **1.38** | **0.72** |
|  | pentadecanoate (15:0) | LC/MS neg | 1361 | [C16537](http://www.genome.jp/dbget-bin/www_bget?cpd%2BC16537) | [HMDB00826](http://www.hmdb.ca/metabolites/HMDB00826) | 13849 | 1.40 | 0.98 | 1.43 |
|  | palmitate (16:0) | LC/MS neg | 1336 | [C00249](http://www.genome.jp/dbget-bin/www_bget?cpd%2BC00249) | [HMDB00220](http://www.hmdb.ca/metabolites/HMDB00220) | 985 | 1.21 | 0.99 | 1.22 |
|  | palmitoleate (16:1n7) | LC/MS neg | 33447 | [C08362](http://www.genome.jp/dbget-bin/www_bget?cpd%2BC08362) | [HMDB03229](http://www.hmdb.ca/metabolites/HMDB03229) | 445638 | 1.19 | 1.01 | 1.18 |
|  | margarate (17:0) | LC/MS neg | 1121 |  | [HMDB02259](http://www.hmdb.ca/metabolites/HMDB02259) | 10465 | 1.41 | 0.91 | 1.55 |
|  | 10-heptadecenoate (17:1n7) | LC/MS neg | 33971 |  | [HMDB60038](http://www.hmdb.ca/metabolites/HMDB60038) | 5312435 | 1.54 | 0.89 | 1.72 |
|  | stearate (18:0) | LC/MS neg | 1358 | [C01530](http://www.genome.jp/dbget-bin/www_bget?cpd%2BC01530) | [HMDB00827](http://www.hmdb.ca/metabolites/HMDB00827) | 5281 | 1.10 | 0.95 | 1.16 |
|  | oleate/vaccenate (18:1) | LC/MS neg | 52285 |  |  |  | 1.16 | 1.04 | 1.11 |
|  | nonadecanoate (19:0) | LC/MS neg | 1356 | [C16535](http://www.genome.jp/dbget-bin/www_bget?cpd%2BC16535) | [HMDB00772](http://www.hmdb.ca/metabolites/HMDB00772) | 12591 | 1.25 | 1.08 | 1.16 |
|  | 10-nonadecenoate (19:1n9) | LC/MS neg | 33972 |  | [HMDB13622](http://www.hmdb.ca/metabolites/HMDB13622) | 5312513 | 1.16 | 0.72 | 1.62 |
|  | arachidate (20:0) | LC/MS neg | 1118 | [C06425](http://www.genome.jp/dbget-bin/www_bget?cpd%2BC06425) | [HMDB02212](http://www.hmdb.ca/metabolites/HMDB02212) | 10467 | 1.34 | 0.92 | 1.45 |
|  | eicosenoate (20:1) | LC/MS neg | 33587 | [C16526](http://www.genome.jp/dbget-bin/www_bget?cpd%2BC16526) | [HMDB02231](http://www.hmdb.ca/metabolites/HMDB02231) | 5282768 | 1.55 | 1.16 | 1.33 |
|  | erucate (22:1n9) | LC/MS neg | 1552 | [C08316](http://www.genome.jp/dbget-bin/www_bget?cpd%2BC08316) | [HMDB02068](http://www.hmdb.ca/metabolites/HMDB02068) | 5281116 | 1.16 | 0.82 | 1.41 |
| Polyunsaturated Fatty Acid (n3 and n6) | hexadecatrienoate (16:3n3) | LC/MS neg | 57651 |  |  | 5312428 | 0.88 | 0.96 | 0.91 |
|  | stearidonate (18:4n3) | LC/MS neg | 33969 | [C16300](http://www.genome.jp/dbget-bin/www_bget?cpd%2BC16300) | [HMDB06547](http://www.hmdb.ca/metabolites/HMDB06547) | 5312508 | 0.89 | 0.66 | 1.34 |
|  | eicosapentaenoate (EPA; 20:5n3) | LC/MS neg | 18467 | [C06428](http://www.genome.jp/dbget-bin/www_bget?cpd%2BC06428) | [HMDB01999](http://www.hmdb.ca/metabolites/HMDB01999) | 446284 | 1.25 | 1.45 | 0.86 |
|  | linoleate (18:2n6) | LC/MS neg | 1105 | [C01595](http://www.genome.jp/dbget-bin/www_bget?cpd%2BC01595) | [HMDB00673](http://www.hmdb.ca/metabolites/HMDB00673) | 5280450 | 1.10 | 0.96 | 1.14 |
|  | linolenate [alpha or gamma; (18:3n3 or 6)] | LC/MS neg | 34035 | [C06426](http://www.genome.jp/dbget-bin/www_bget?cpd%2BC06426) | [HMDB03073](http://www.hmdb.ca/metabolites/HMDB03073) | 5280934 | 1.08 | 0.97 | 1.11 |
|  | dihomo-linolenate (20:3n3 or n6) | LC/MS neg | 35718 | [C03242](http://www.genome.jp/dbget-bin/www_bget?cpd%2BC03242) | [HMDB02925](http://www.hmdb.ca/metabolites/HMDB02925) | 5280581 | 0.80 | 0.87 | 0.92 |
|  | arachidonate (20:4n6) | LC/MS neg | 1110 | [C00219](http://www.genome.jp/dbget-bin/www_bget?cpd%2BC00219) | [HMDB01043](http://www.hmdb.ca/metabolites/HMDB01043) | 444899 | 1.30 | 1.19 | 1.10 |
|  | dihomo-linoleate (20:2n6) | LC/MS neg | 17805 | [C16525](http://www.genome.jp/dbget-bin/www_bget?cpd%2BC16525) | [HMDB05060](http://www.hmdb.ca/metabolites/HMDB05060) | 6439848 | 1.61 | 1.87 | 0.86 |
| Fatty Acid, Branched | 13-methylmyristate (i15:0) | LC/MS neg | 38293 |  |  | 151014 | 1.20 | 0.86 | 1.40 |
|  | 15-methylpalmitate (i17:0) | LC/MS neg | 38768 |  |  | 17903417 | 1.18 | 0.77 | 1.54 |
|  | 17-methylstearate (i19:0) | LC/MS neg | 38296 |  | [HMDB37397](http://www.hmdb.ca/metabolites/HMDB37397) | 3083779 | 1.27 | 0.80 | 1.59 |
| Fatty Acid, Dicarboxylate | glutarate (C5-DC) | LC/MS polar | 396 | [C00489](http://www.genome.jp/dbget-bin/www_bget?cpd%2BC00489) | [HMDB00661](http://www.hmdb.ca/metabolites/HMDB00661) | 743 | 0.90 | 1.00 | 0.90 |
|  | 2-hydroxyglutarate | LC/MS pos early | 37253 | [C02630](http://www.genome.jp/dbget-bin/www_bget?cpd%2BC02630) | [HMDB00606](http://www.hmdb.ca/metabolites/HMDB00606) | 43 | **1.10** | 1.04 | 1.06 |
|  | adipate (C6-DC) | LC/MS polar | 21134 | [C06104](http://www.genome.jp/dbget-bin/www_bget?cpd%2BC06104) | [HMDB00448](http://www.hmdb.ca/metabolites/HMDB00448) | 196 | 0.90 | **1.38** | **0.65** |
|  | suberate (C8-DC) | LC/MS polar | 15730 | [C08278](http://www.genome.jp/dbget-bin/www_bget?cpd%2BC08278) | [HMDB00893](http://www.hmdb.ca/metabolites/HMDB00893) | 10457 | 1.46 | **1.51** | 0.97 |
|  | azelate (C9-DC) | LC/MS neg | 18362 | [C08261](http://www.genome.jp/dbget-bin/www_bget?cpd%2BC08261) | [HMDB00784](http://www.hmdb.ca/metabolites/HMDB00784) | 2266 | 2.01 | 2.14 | 0.94 |
|  | sebacate (C10-DC) | LC/MS polar | 32398 | [C08277](http://www.genome.jp/dbget-bin/www_bget?cpd%2BC08277) | [HMDB00792](http://www.hmdb.ca/metabolites/HMDB00792) | 5192 | **0.72** | 0.94 | **0.76** |
|  | dodecanedioate (C12-DC) | LC/MS neg | 32388 | [C02678](http://www.genome.jp/dbget-bin/www_bget?cpd%2BC02678) | [HMDB00623](http://www.hmdb.ca/metabolites/HMDB00623) | 12736 | **0.59** | 0.94 | **0.62** |
|  | tetradecanedioate (C14-DC) | LC/MS neg | 35669 |  | [HMDB00872](http://www.hmdb.ca/metabolites/HMDB00872) | 13185 | 0.92 | 1.04 | 0.89 |
|  | hexadecanedioate (C16-DC) | LC/MS neg | 35678 | [C19615](http://www.genome.jp/dbget-bin/www_bget?cpd%2BC19615) | [HMDB00672](http://www.hmdb.ca/metabolites/HMDB00672) | 10459 | 1.30 | 1.18 | 1.10 |
|  | hexadecenedioate (C16:1-DC)* | LC/MS neg | 61862 |  |  |  | 1.09 | 1.32 | 0.82 |
|  | octadecadienedioate (C18:2-DC)* | LC/MS neg | 61860 |  |  |  | 1.00 | 1.00 | 1.00 |

| **Sub Pathway** | **Biochemical Name** | **Platform** | **Comp ID** | **KEGG** | **HMDB** | **PubChem** | **Gal4>UAS**  **GAL4 Ctrl** | **Gal4>UAS**  **UAS Ctrl** | **UAS Ctrl GAL4 Ctrl** |
| --- | --- | --- | --- | --- | --- | --- | --- | --- | --- |
| Fatty Acid, Amino | 2-aminooctanoate | LC/MS pos late | 43343 |  | [HMDB00991](http://www.hmdb.ca/metabolites/HMDB00991) | 69522 | **2.29** | 0.97 | **2.36** |
|  | N-acetyl-2-aminooctanoate* | LC/MS neg | 62059 |  | [HMDB59745](http://www.hmdb.ca/metabolites/HMDB59745) | 95555 | 1.15 | 0.90 | 1.28 |
| Fatty Acid Metabolism (also BCAA Metabolism) | propionylglycine | LC/MS polar | 31932 |  | [HMDB00783](http://www.hmdb.ca/metabolites/HMDB00783) | 98681 | **0.65** | 1.12 | **0.58** |
|  | methylmalonate (MMA) | LC/MS polar | 1496 | [C02170](http://www.genome.jp/dbget-bin/www_bget?cpd%2BC02170) | [HMDB00202](http://www.hmdb.ca/metabolites/HMDB00202) | 487 | **0.70** | 0.96 | **0.73** |
| Fatty Acid Metabolism(Acyl Glycine) | hexanoylglycine | LC/MS neg | 35436 |  | [HMDB00701](http://www.hmdb.ca/metabolites/HMDB00701) | 99463 | 1.19 | 1.10 | 1.08 |
| Fatty Acid Metabolism(Acyl Carnitine) | acetylcarnitine (C2) | LC/MS pos early | 32198 | [C02571](http://www.genome.jp/dbget-bin/www_bget?cpd%2BC02571) | [HMDB00201](http://www.hmdb.ca/metabolites/HMDB00201) | 1 | **4.25** | **2.68** | **1.59** |
|  | myristoylcarnitine (C14) | LC/MS pos late | 33952 |  | [HMDB05066](http://www.hmdb.ca/metabolites/HMDB05066) | 6426854 | **28.81** | **26.39** | 1.09 |
|  | palmitoylcarnitine (C16) | LC/MS pos late | 44681 | [C02990](http://www.genome.jp/dbget-bin/www_bget?cpd%2BC02990) | [HMDB00222](http://www.hmdb.ca/metabolites/HMDB00222) | 461 | **4.52** | **1.74** | **2.60** |
|  | palmitoleoylcarnitine (C16:1)* | LC/MS pos late | 53223 |  |  | 71464547 | **26.50** | **10.35** | **2.56** |
|  | stearoylcarnitine (C18) | LC/MS pos late | 34409 |  | [HMDB00848](http://www.hmdb.ca/metabolites/HMDB00848) | 6426855 | **1.43** | 1.06 | **1.34** |
|  | linoleoylcarnitine (C18:2)* | LC/MS pos late | 46223 |  | [HMDB06469](http://www.hmdb.ca/metabolites/HMDB06469) | 6450015 | **6.77** | **3.14** | **2.15** |
|  | oleoylcarnitine (C18:1) | LC/MS pos late | 35160 |  | [HMDB05065](http://www.hmdb.ca/metabolites/HMDB05065) | 6441392 | **4.00** | **1.92** | **2.08** |
|  | arachidoylcarnitine (C20)* | LC/MS pos late | 57513 |  | [HMDB06460](http://www.hmdb.ca/metabolites/HMDB06460) |  | 1.28 | 1.17 | 1.09 |
|  | behenoylcarnitine (C22)* | LC/MS pos late | 57514 |  |  |  | **1.31** | 1.09 | **1.20** |
|  | eicosenoylcarnitine (C20:1)* | LC/MS pos late | 57519 |  |  |  | **2.28** | 1.24 | **1.84** |
|  | lignoceroylcarnitine (C24)* | LC/MS pos late | 57515 |  |  |  | **0.72** | **0.83** | **0.87** |
|  | margaroylcarnitine (C17)* | LC/MS pos late | 57512 |  | [HMDB06210](http://www.hmdb.ca/metabolites/HMDB06210) |  | **4.05** | **1.47** | **2.76** |
|  | nervonoylcarnitine (C24:1)* | LC/MS pos late | 57531 |  |  |  | **2.36** | 1.26 | **1.87** |
|  | cerotoylcarnitine (C26)* | LC/MS pos late | 57516 |  | [HMDB06347](http://www.hmdb.ca/metabolites/HMDB06347) |  | 0.85 | 0.97 | 0.87 |
|  | ximenoylcarnitine (C26:1)* | LC/MS pos late | 57517 |  |  |  | **0.75** | 0.88 | 0.85 |
| Carnitine Metabolism | deoxycarnitine | LC/MS pos early | 36747 | [C01181](http://www.genome.jp/dbget-bin/www_bget?cpd%2BC01181) | [HMDB01161](http://www.hmdb.ca/metabolites/HMDB01161) | 134 | 1.77 | 1.17 | 1.52 |
|  | carnitine | LC/MS pos early | 15500 | [C00318](http://www.genome.jp/dbget-bin/www_bget?cpd%2BC00318) | [HMDB00062](http://www.hmdb.ca/metabolites/HMDB00062) | 10917 | **1.62** | 1.01 | **1.59** |
| Fatty Acid, Monohydroxy | 3-hydroxypropanoate | LC/MS polar | 1556 | [C01013](http://www.genome.jp/dbget-bin/www_bget?cpd%2BC01013) | [HMDB00700](http://www.hmdb.ca/metabolites/HMDB00700) | 68152 | 0.96 | 1.20 | 0.80 |
|  | 3-hydroxydecanoate | LC/MS neg | 22053 |  | [HMDB02203](http://www.hmdb.ca/metabolites/HMDB02203) | 26612 | 0.90 | 0.71 | 1.25 |
|  | 3-hydroxysebacate | LC/MS polar | 31943 |  | [HMDB00350](http://www.hmdb.ca/metabolites/HMDB00350) | 3017884 | 0.62 | 0.57 | 1.09 |
|  | 3-hydroxylaurate | LC/MS neg | 32457 |  | [HMDB00387](http://www.hmdb.ca/metabolites/HMDB00387) | 94216 | 1.07 | 1.30 | 0.82 |
|  | 3-hydroxymyristate | LC/MS neg | 21158 |  |  | 16064 | **1.63** | 1.02 | **1.59** |
|  | 3-hydroxyoleate* | LC/MS neg | 61843 |  |  |  | 1.51 | 0.88 | 1.72 |
|  | 13-HODE + 9-HODE | LC/MS neg | 37752 |  |  | 43013 | 1.19 | 1.05 | 1.13 |
| Fatty Acid, Dihydroxy | 12,13-DiHOME | LC/MS neg | 38395 | [C14829](http://www.genome.jp/dbget-bin/www_bget?cpd%2BC14829) | [HMDB04705](http://www.hmdb.ca/metabolites/HMDB04705) | 10236635 | 0.63 | 0.99 | 0.63 |
|  | 9,10-DiHOME | LC/MS neg | 38399 | [C14828](http://www.genome.jp/dbget-bin/www_bget?cpd%2BC14828) | [HMDB04704](http://www.hmdb.ca/metabolites/HMDB04704) | 9966640 | 0.83 | 1.37 | **0.61** |
| Endocannabinoid | N-myristoyltaurine* | LC/MS neg | 61825 |  |  | 3810823 | 1.24 | 0.63 | **1.97** |
|  | N-oleoyltaurine | LC/MS neg | 39732 |  |  | 6437033 | 1.16 | 0.81 | 1.42 |
|  | N-palmitoleoyltaurine* | LC/MS neg | 61824 |  |  |  | 0.97 | 0.82 | 1.18 |
|  | linoleoyl ethanolamide | LC/MS neg | 52608 |  | [HMDB12252](http://www.hmdb.ca/metabolites/HMDB12252) | 5283446 | 1.02 | 0.73 | 1.40 |
| Inositol Metabolism | myo-inositol | LC/MS polar | 1124 | [C00137](http://www.genome.jp/dbget-bin/www_bget?cpd%2BC00137) | [HMDB00211](http://www.hmdb.ca/metabolites/HMDB00211) | 892 | 1.03 | 0.94 | 1.10 |
| Phospholipid Metabolism | choline | LC/MS pos early | 15506 | [C00114](http://www.genome.jp/dbget-bin/www_bget?cpd%2BC00114) | [HMDB00097](http://www.hmdb.ca/metabolites/HMDB00097) | 305 | 1.01 | 1.04 | 0.97 |
|  | choline phosphate | LC/MS pos early | 34396 | [C00588](http://www.genome.jp/dbget-bin/www_bget?cpd%2BC00588) | [HMDB01565](http://www.hmdb.ca/metabolites/HMDB01565) | 1014 | 1.08 | **1.14** | 0.95 |
|  | cytidine 5'-diphosphocholine | LC/MS pos early | 34418 | [C00307](http://www.genome.jp/dbget-bin/www_bget?cpd%2BC00307) | [HMDB01413](http://www.hmdb.ca/metabolites/HMDB01413) | 13804 | 1.13 | 0.92 | 1.23 |
|  | glycerophosphorylcholine (GPC) | LC/MS pos early | 15990 | [C00670](http://www.genome.jp/dbget-bin/www_bget?cpd%2BC00670) | [HMDB00086](http://www.hmdb.ca/metabolites/HMDB00086) | 71920 | 0.97 | 0.99 | 0.97 |
|  | phosphoethanolamine | LC/MS pos early | 1600 | [C00346](http://www.genome.jp/dbget-bin/www_bget?cpd%2BC00346) | [HMDB00224](http://www.hmdb.ca/metabolites/HMDB00224) | 1015 | 0.93 | 1.05 | 0.88 |
|  | cytidine-5'-diphosphoethanolamine | LC/MS polar | 34410 | [C00570](http://www.genome.jp/dbget-bin/www_bget?cpd%2BC00570) | [HMDB01564](http://www.hmdb.ca/metabolites/HMDB01564) | 123727 | 0.97 | **0.85** | **1.14** |
|  | glycerophosphoethanolamine | LC/MS polar | 37455 | [C01233](http://www.genome.jp/dbget-bin/www_bget?cpd%2BC01233) | [HMDB00114](http://www.hmdb.ca/metabolites/HMDB00114) | 123874 | 0.89 | 0.97 | 0.91 |
|  | glycerophosphoserine* | LC/MS pos early | 57404 |  |  | 3081457 | 1.33 | 1.48 | 0.90 |
|  | glycerophosphoinositol* | LC/MS pos early | 52307 |  |  | 167572 | 1.23 | 1.31 | 0.93 |
| Phosphatidylcholine (PC) | 1-myristoyl-2-palmitoyl-GPC (14:0/16:0) | LC/MS pos late | 19258 |  | [HMDB07869](http://www.hmdb.ca/metabolites/HMDB07869) | 129657 | **0.79** | 0.92 | 0.87 |
|  | 1,2-dipalmitoyl-GPC (16:0/16:0) | LC/MS pos late | 19130 |  | [HMDB00564](http://www.hmdb.ca/metabolites/HMDB00564) | 452110 | **0.71** | **0.90** | **0.79** |
|  | 1-palmitoyl-2-palmitoleoyl-GPC (16:0/16:1)* | LC/MS pos late | 52470 |  | [HMDB07969](http://www.hmdb.ca/metabolites/HMDB07969) |  | 1.04 | 0.92 | **1.12** |
|  | 1-palmitoyl-2-stearoyl-GPC (16:0/18:0) | LC/MS pos late | 52616 |  | [HMDB07970](http://www.hmdb.ca/metabolites/HMDB07970) |  | **0.71** | 0.92 | 0.77 |
|  | 1-palmitoyl-2-oleoyl-GPC (16:0/18:1) | LC/MS pos late | 52461 |  | [HMDB07972](http://www.hmdb.ca/metabolites/HMDB07972) | 6436017 | 0.95 | 0.97 | 0.98 |
|  | 1,2-dipalmitoleoyl-GPC (16:1/16:1)* | LC/MS pos late | 52472 |  |  |  | 0.95 | 0.83 | 1.15 |
|  | 1-palmitoleoyl-2-linolenoyl-GPC (16:1/18:3)* | LC/MS pos late | 53180 |  | [HMDB08008](http://www.hmdb.ca/metabolites/HMDB08008) |  | 0.92 | 1.03 | 0.90 |
|  | 1,2-distearoyl-GPC (18:0/18:0) | LC/MS pos late | 19132 |  | [HMDB08036](http://www.hmdb.ca/metabolites/HMDB08036) | 94190 | **0.72** | 0.93 | 0.78 |
|  | 1-stearoyl-2-oleoyl-GPC (18:0/18:1) | LC/MS pos late | 52438 |  | [HMDB08038](http://www.hmdb.ca/metabolites/HMDB08038) |  | 0.85 | 1.01 | 0.84 |
|  | 1-oleoyl-2-linoleoyl-GPC (18:1/18:2)* | LC/MS pos late | 52453 |  |  |  | 0.90 | 0.88 | 1.02 |
|  | 1,2-dilinoleoyl-GPC (18:2/18:2) | LC/MS pos late | 52603 |  | [HMDB08138](http://www.hmdb.ca/metabolites/HMDB08138) | 5288075 | 0.78 | 0.81 | 0.97 |
|  | 1-linoleoyl-2-linolenoyl-GPC (18:2/18:3)* | LC/MS pos late | 53176 |  | [HMDB08141](http://www.hmdb.ca/metabolites/HMDB08141) |  | 0.79 | 0.88 | 0.90 |
|  | 1,2-dilinolenoyl-GPC (18:3/18:3)* | LC/MS pos late | 53179 |  | [HMDB08206](http://www.hmdb.ca/metabolites/HMDB08206) |  | 0.77 | 0.89 | 0.87 |
| Phosphatidylethanolamine (PE) | 1,2-dipalmitoyl-GPE (16:0/16:0)* | LC/MS pos late | 57341 |  | [HMDB08923](http://www.hmdb.ca/metabolites/HMDB08923) | 445468 | **0.84** | 0.97 | **0.87** |
|  | 1-palmitoyl-2-stearoyl-GPE (16:0/18:0)* | LC/MS pos late | 57388 |  | [HMDB08925](http://www.hmdb.ca/metabolites/HMDB08925) | 5326793 | **0.64** | 0.78 | 0.82 |
|  | 1-palmitoyl-2-oleoyl-GPE (16:0/18:1) | LC/MS pos late | 19263 |  | [HMDB05320](http://www.hmdb.ca/metabolites/HMDB05320) | 5283496 | 0.96 | 0.94 | 1.02 |
|  | 1,2-dipalmitoleoyl-GPE (16:1/16:1)* | LC/MS pos late | 52688 |  | [HMDB05342](http://www.hmdb.ca/metabolites/HMDB05342) | 9546809 | 0.84 | 0.86 | 0.98 |
|  | 1-stearoyl-2-oleoyl-GPE (18:0/18:1) | LC/MS pos late | 42448 |  | [HMDB08993](http://www.hmdb.ca/metabolites/HMDB08993) |  | 0.96 | 1.03 | 0.93 |
|  | 1-oleoyl-2-linoleoyl-GPE (18:1/18:2)* | LC/MS pos late | 52687 |  | [HMDB05349](http://www.hmdb.ca/metabolites/HMDB05349) | 9546753 | 0.93 | 0.91 | 1.03 |
|  | 1,2-dilinoleoyl-GPE (18:2/18:2)* | LC/MS pos late | 53174 |  | [HMDB09093](http://www.hmdb.ca/metabolites/HMDB09093) | 9546812 | 0.85 | 0.85 | 0.99 |
| Phosphatidylserine (PS) | 1,2-dioleoyl-GPS (18:1/18:1) | LC/MS pos late | 19191 |  |  | 6438639 | 1.29 | 0.95 | **1.35** |
| Phosphatidylglycerol (PG) | 1-palmitoyl-2-palmitoleoyl-GPG (16:0/16:1)* | LC/MS pos late | 53213 |  |  |  | 1.32 | 0.78 | **1.68** |
|  | 1-palmitoyl-2-oleoyl-GPG (16:0/18:1) | LC/MS pos late | 52448 |  |  | 5283509 | 0.91 | 0.81 | 1.11 |
| Phosphatidylinositol (PI) | 1,2-dipalmitoleoyl-GPI (16:1/16:1)* | LC/MS pos late | 52721 |  |  |  | 1.06 | 0.90 | 1.18 |
|  | 1-palmitoyl-2-oleoyl-GPI (16:0/18:1)* | LC/MS polar | 52669 |  | [HMDB09783](http://www.hmdb.ca/metabolites/HMDB09783) |  | 0.91 | **0.77** | **1.18** |
|  | 1-oleoyl-2-linoleoyl-GPI (18:1/18:2)* | LC/MS polar | 52451 |  | [HMDB09838](http://www.hmdb.ca/metabolites/HMDB09838) |  | 0.89 | 0.90 | 0.99 |

| **Sub Pathway** | **Biochemical Name** | **Platform** | **Comp ID** | **KEGG** | **HMDB** | **PubChem** | **Gal4>UAS**  **GAL4 Ctrl** | **Gal4>UAS**  **UAS Ctrl** | **UAS Ctrl GAL4 Ctrl** |
| --- | --- | --- | --- | --- | --- | --- | --- | --- | --- |
| Lysophospholipid | 1-linolenoyl-GPG (18:3)* | LC/MS neg | 62368 |  |  |  | 1.17 | 0.98 | 1.20 |
|  | 1-palmitoyl-GPC (16:0) | LC/MS pos late | 33955 |  | [HMDB10382](http://www.hmdb.ca/metabolites/HMDB10382) | 86554 | **0.79** | 0.98 | **0.81** |
|  | 2-palmitoyl-GPC (16:0)* | LC/MS pos late | 35253 |  | [HMDB61702](http://www.hmdb.ca/metabolites/HMDB61702) | 15061532 | 0.98 | 0.92 | 1.07 |
|  | 1-palmitoleoyl-GPC (16:1)* | LC/MS pos late | 33230 |  | [HMDB10383](http://www.hmdb.ca/metabolites/HMDB10383) | 24779461 | 1.06 | 1.07 | 0.99 |
|  | 2-palmitoleoyl-GPC (16:1)* | LC/MS pos late | 35819 |  | [HMDB10383](http://www.hmdb.ca/metabolites/HMDB10383) |  | 1.14 | 0.99 | 1.15 |
|  | 1-stearoyl-GPC (18:0) | LC/MS pos late | 33961 |  | [HMDB10384](http://www.hmdb.ca/metabolites/HMDB10384) | 497299 | 0.75 | 1.01 | 0.75 |
|  | 1-oleoyl-GPC (18:1) | LC/MS pos late | 48258 |  | [HMDB02815](http://www.hmdb.ca/metabolites/HMDB02815) | 16081932 | 1.06 | 1.07 | 0.99 |
|  | 1-linoleoyl-GPC (18:2) | LC/MS neg | 34419 | [C04100](http://www.genome.jp/dbget-bin/www_bget?cpd%2BC04100) | [HMDB10386](http://www.hmdb.ca/metabolites/HMDB10386) | 11988421 | 1.15 | 0.95 | 1.20 |
|  | 1-linolenoyl-GPC (18:3)* | LC/MS pos late | 45951 |  | [HMDB10388](http://www.hmdb.ca/metabolites/HMDB10388) |  | **0.74** | 0.98 | **0.75** |
|  | 1-palmitoyl-GPE (16:0) | LC/MS pos late | 35631 |  | [HMDB11503](http://www.hmdb.ca/metabolites/HMDB11503) | 9547069 | 0.85 | 0.93 | 0.92 |
|  | 1-stearoyl-GPE (18:0) | LC/MS pos late | 42398 |  | [HMDB11130](http://www.hmdb.ca/metabolites/HMDB11130) | 9547068 | 0.76 | 0.91 | 0.84 |
|  | 2-stearoyl-GPE (18:0)* | LC/MS neg | 41220 |  | [HMDB11129](http://www.hmdb.ca/metabolites/HMDB11129) |  | 0.90 | 1.05 | 0.86 |
|  | 1-oleoyl-GPE (18:1) | LC/MS pos late | 35628 |  | [HMDB11506](http://www.hmdb.ca/metabolites/HMDB11506) | 9547071 | 0.98 | 0.94 | 1.04 |
|  | 1-linoleoyl-GPE (18:2)* | LC/MS pos late | 36600 |  | [HMDB11507](http://www.hmdb.ca/metabolites/HMDB11507) | 52925130 | 0.95 | 1.00 | 0.95 |
|  | 1-palmitoyl-GPS (16:0)* | LC/MS neg | 46130 |  |  | 9547100 | 1.57 | 1.17 | 1.35 |
|  | 1-stearoyl-GPS (18:0)* | LC/MS neg | 45966 |  |  | 9547101 | 1.23 | 0.87 | 1.42 |
|  | 1-oleoyl-GPS (18:1) | LC/MS neg | 19260 |  | [HMDB61694](http://www.hmdb.ca/metabolites/HMDB61694) | 9547099 | 1.49 | 1.00 | 1.49 |
|  | 1-linoleoyl-GPS (18:2)* | LC/MS neg | 43676 |  |  |  | 1.11 | 0.81 | 1.38 |
|  | 1-palmitoyl-GPG (16:0)* | LC/MS neg | 45970 |  |  | 3300276 | 1.03 | 0.77 | 1.34 |
|  | 1-stearoyl-GPG (18:0) | LC/MS neg | 34437 |  |  |  | 0.96 | 0.93 | 1.03 |
|  | 1-oleoyl-GPG (18:1)* | LC/MS neg | 45968 |  |  |  | 1.27 | 1.16 | 1.09 |
|  | 1-linoleoyl-GPG (18:2)* | LC/MS neg | 54885 |  |  |  | 1.11 | 0.91 | 1.22 |
|  | 1-palmitoyl-GPI (16:0) | LC/MS neg | 35305 |  | [HMDB61695](http://www.hmdb.ca/metabolites/HMDB61695) |  | 1.11 | 0.95 | 1.17 |
|  | 1-stearoyl-GPI (18:0) | LC/MS neg | 19324 |  | [HMDB61696](http://www.hmdb.ca/metabolites/HMDB61696) |  | 0.83 | 0.66 | 1.26 |
|  | 1-oleoyl-GPI (18:1)* | LC/MS neg | 36602 |  |  |  | 1.65 | 1.12 | 1.48 |
|  | 1-linoleoyl-GPI (18:2)* | LC/MS neg | 36594 |  |  |  | 1.20 | 0.99 | 1.21 |
| Glycolipid Metabolism | 1,2-dilinoleoyl-galactosylglycerol (18:2/18:2)* | LC/MS pos late | 54899 |  |  | 6535011 | 1.05 | 0.90 | 1.16 |
| Plasmalogen | 1-(1-enyl-palmitoyl)-2-oleoyl-GPE (P-16:0/18:1)* | LC/MS pos late | 52477 |  | [HMDB11342](http://www.hmdb.ca/metabolites/HMDB11342) |  | 1.23 | 1.06 | 1.15 |
|  | 1-(1-enyl-stearoyl)-2-oleoyl-GPE (P-18:0/18:1) | LC/MS pos late | 52614 |  | [HMDB11375](http://www.hmdb.ca/metabolites/HMDB11375) |  | 0.94 | 0.86 | 1.09 |
|  | 1-(1-enyl-stearoyl)-2-linoleoyl-GPE (P-18:0/18:2)* | LC/MS pos late | 52748 |  | [HMDB11376](http://www.hmdb.ca/metabolites/HMDB11376) |  | 0.75 | 1.02 | 0.74 |
| Lysoplasmalogen | 1-(1-enyl-stearoyl)-GPE (P-18:0)* | LC/MS pos late | 39271 |  |  |  | 0.67 | 1.01 | 0.67 |
| Glycerolipid Metabolism | glycerol | LC/MS neg | 15122 | [C00116](http://www.genome.jp/dbget-bin/www_bget?cpd%2BC00116) | [HMDB00131](http://www.hmdb.ca/metabolites/HMDB00131) | 753 | 1.19 | 1.09 | 1.09 |
|  | glycerol 3-phosphate | LC/MS polar | 43847 | [C00093](http://www.genome.jp/dbget-bin/www_bget?cpd%2BC00093) | [HMDB00126](http://www.hmdb.ca/metabolites/HMDB00126) | 754 | 1.09 | 1.15 | 0.95 |
|  | glycerophosphoglycerol | LC/MS polar | 48857 | [C03274](http://www.genome.jp/dbget-bin/www_bget?cpd%2BC03274) |  | 439964 | 1.02 | 1.26 | 0.81 |
| Monoacylglycerol | 1-myristoylglycerol (14:0) | LC/MS neg | 35625 | [C01885](http://www.genome.jp/dbget-bin/www_bget?cpd%2BC01885) | [HMDB11561](http://www.hmdb.ca/metabolites/HMDB11561) | 79050 | 1.26 | 0.76 | 1.65 |
|  | 1-palmitoylglycerol (16:0) | LC/MS neg | 21127 |  | [HMDB31074](http://www.hmdb.ca/metabolites/HMDB31074) | 14900 | 1.04 | 0.93 | 1.12 |
|  | 1-palmitoleoylglycerol (16:1)* | LC/MS neg | 52431 |  | [HMDB11565](http://www.hmdb.ca/metabolites/HMDB11565) |  | 2.31 | 1.29 | 1.79 |
|  | 1-oleoylglycerol (18:1) | LC/MS neg | 21184 |  | [HMDB11567](http://www.hmdb.ca/metabolites/HMDB11567) | 5283468 | 1.85 | 1.31 | 1.41 |
|  | 1-linoleoylglycerol (18:2) | LC/MS neg | 27447 |  |  | 5283469 | 1.95 | 1.40 | 1.40 |
|  | 2-myristoylglycerol (14:0) | LC/MS neg | 34383 |  | [HMDB11530](http://www.hmdb.ca/metabolites/HMDB11530) | 137938 | 1.06 | 0.75 | 1.41 |
|  | 2-palmitoylglycerol (16:0) | LC/MS neg | 33419 |  | [HMDB11533](http://www.hmdb.ca/metabolites/HMDB11533) | 123409 | 1.49 | 1.09 | 1.37 |
|  | 2-palmitoleoylglycerol (16:1)* | LC/MS neg | 52432 |  | [HMDB11565](http://www.hmdb.ca/metabolites/HMDB11565) |  | 2.25 | 1.15 | 1.96 |
|  | 2-oleoylglycerol (18:1) | LC/MS neg | 21232 |  | [HMDB11537](http://www.hmdb.ca/metabolites/HMDB11537) | 5319879 | 1.66 | 0.87 | 1.90 |
|  | 2-linoleoylglycerol (18:2) | LC/MS neg | 32506 |  | [HMDB11538](http://www.hmdb.ca/metabolites/HMDB11538) | 5365676 | 1.61 | 1.64 | 0.98 |
| Diacylglycerol | diacylglycerol (12:0/18:1, 14:0/16:1, 16:0/14:1) [2]* | LC/MS pos late | 55001 |  |  |  | 1.23 | 1.19 | 1.03 |
|  | diacylglycerol (14:0/18:1, 16:0/16:1) [2]* | LC/MS pos late | 54954 |  |  |  | **1.39** | 1.07 | 1.30 |
|  | diacylglycerol (16:1/18:2 [2], 16:0/18:3 [1])* | LC/MS pos late | 54966 |  |  |  | 1.18 | 1.10 | 1.07 |
|  | palmitoyl-oleoyl-glycerol (16:0/18:1) [2]* | LC/MS pos late | 54942 | [C13861](http://www.genome.jp/dbget-bin/www_bget?cpd%2BC13861) | [HMDB07102](http://www.hmdb.ca/metabolites/HMDB07102) |  | 1.45 | 0.93 | 1.56 |
|  | palmitoyl-linoleoyl-glycerol (16:0/18:2) [2]* | LC/MS pos late | 52634 |  | [HMDB07103](http://www.hmdb.ca/metabolites/HMDB07103) |  | 1.55 | 0.93 | 1.67 |
|  | palmitoleoyl-oleoyl-glycerol (16:1/18:1) [2]* | LC/MS pos late | 52631 |  |  |  | 1.12 | 1.07 | 1.04 |
|  | palmitoleoyl-linoleoyl-glycerol (16:1/18:2) [1]* | LC/MS pos late | 54967 |  | [HMDB07132](http://www.hmdb.ca/metabolites/HMDB07132) |  | 1.22 | 1.13 | 1.08 |
|  | oleoyl-oleoyl-glycerol (18:1/18:1) [2]* | LC/MS pos late | 54946 |  | [HMDB07218](http://www.hmdb.ca/metabolites/HMDB07218) |  | 1.08 | 1.04 | 1.03 |
|  | oleoyl-linoleoyl-glycerol (18:1/18:2) [2] | LC/MS pos late | 46799 |  | [HMDB07219](http://www.hmdb.ca/metabolites/HMDB07219) |  | 1.00 | 1.17 | 0.86 |
|  | oleoyl-linolenoyl-glycerol (18:1/18:3) [2]* | LC/MS pos late | 54970 |  | [HMDB07220](http://www.hmdb.ca/metabolites/HMDB07220) |  | 0.93 | **1.81** | **0.51** |
|  | linoleoyl-linolenoyl-glycerol (18:2/18:3) [2]* | LC/MS pos late | 54964 |  | [HMDB07250](http://www.hmdb.ca/metabolites/HMDB07250) |  | **0.70** | 1.22 | **0.57** |
| Sphingolipid Synthesis | sphinganine | LC/MS pos late | 17769 | [C00836](http://www.genome.jp/dbget-bin/www_bget?cpd%2BC00836) | [HMDB00269](http://www.hmdb.ca/metabolites/HMDB00269) | 3126 | **0.55** | 1.00 | **0.55** |
|  | tetradecasphinganine (d14:0)* | LC/MS pos late | 57543 |  |  |  | **1.15** | 1.08 | 1.07 |
|  | hexadecasphinganine (d16:0)* | LC/MS pos late | 57544 | [C13915](http://www.genome.jp/dbget-bin/www_bget?cpd%2BC13915) |  | 656816 | 1.21 | 1.04 | 1.16 |
| Dihydroceramides | N-arachidoyl-tetradecanoylsphinganine (d14:0/20:0)* | LC/MS pos late | 57499 |  |  |  | 0.89 | 1.07 | **0.84** |
|  | N-behenoyl-tetradecanoylsphinganine (d14:0/22:0)* | LC/MS pos late | 57501 |  |  |  | 0.81 | 0.94 | 0.86 |
| Ceramides | N-stearoyl-tetradecanoylsphingosine (d14:1/18:0)* | LC/MS pos late | 57500 |  |  |  | 1.02 | 1.09 | 0.94 |
|  | N-arachidoyl-tetradecanoylsphingosine (d14:1/20:0)* | LC/MS pos late | 57505 |  |  |  | 0.95 | 1.17 | **0.81** |
|  | N-behenoyl-tetradecanoylsphingosine (d14:1/22:0)* | LC/MS pos late | 57494 |  |  |  | 0.94 | 0.97 | 0.97 |
| Hexosylceramides (HCER) | glycosyl-N-arachidoyl-tetradecanoylsphingosine (d14:1/20:0)* | LC/MS pos late | 57496 |  |  |  | **1.33** | 1.04 | **1.28** |
|  | glycosyl-N-behenoyl-tetradecasphingosine (d14:1/22:0)* | LC/MS pos late | 57849 |  |  |  | **2.56** | 0.90 | **2.84** |
|  | glycosyl ceramide (d14:1/24:0, d16:1/22:0)* | LC/MS pos late | 57495 |  |  |  | **5.01** | **0.70** | **7.14** |
| Lactosylceramides (LCER) | lactosyl-N-arachidoyl-tetradecanoylsphingosine (d14:1/20:0)* | LC/MS pos late | 57498 |  |  |  | **1.44** | **1.44** | 1.00 |
| Sphingosines | tetradecanoylsphingosine (d14:1)* | LC/MS pos late | 57493 |  |  |  | 1.15 | 0.98 | **1.17** |
|  | hexadecasphingosine (d16:1)* | LC/MS pos late | 57428 |  |  |  | 1.04 | 1.03 | 1.01 |
| Mevalonate Metabolism | 3-hydroxy-3-methylglutarate | LC/MS polar | 531 | [C03761](http://www.genome.jp/dbget-bin/www_bget?cpd%2BC03761) | [HMDB00355](http://www.hmdb.ca/metabolites/HMDB00355) | 1662 | 1.07 | 1.08 | 1.00 |
|  | mevalonate | LC/MS polar | 39583 | [C02104](http://www.genome.jp/dbget-bin/www_bget?cpd%2BC02104) | [HMDB00227](http://www.hmdb.ca/metabolites/HMDB00227) | 439230 | 0.89 | 1.91 | 0.46 |
| Sterol | beta-sitosterol | LC/MS pos late | 27414 | [C01753](http://www.genome.jp/dbget-bin/www_bget?cpd%2BC01753) | [HMDB00852](http://www.hmdb.ca/metabolites/HMDB00852) | 222284 | 1.08 | 1.14 | 0.94 |
|  | campesterol | LC/MS pos late | 33997 | [C01789](http://www.genome.jp/dbget-bin/www_bget?cpd%2BC01789) | [HMDB02869](http://www.hmdb.ca/metabolites/HMDB02869) | 173183 | 1.00 | 1.19 | 0.84 |
|  | ergosterol | LC/MS pos late | 27553 | [C01694](http://www.genome.jp/dbget-bin/www_bget?cpd%2BC01694) | [HMDB00878](http://www.hmdb.ca/metabolites/HMDB00878) | 444679 | 0.93 | 0.99 | 0.94 |
| Purine Metabolism, (Hypo)Xanthine/Inosine containing | inosine 5'-monophosphate (IMP) | LC/MS pos early | 2133 | [C00130](http://www.genome.jp/dbget-bin/www_bget?cpd%2BC00130) | [HMDB00175](http://www.hmdb.ca/metabolites/HMDB00175) | 8582 | 1.19 | 0.70 | **1.70** |
|  | inosine | LC/MS neg | 1123 | [C00294](http://www.genome.jp/dbget-bin/www_bget?cpd%2BC00294) | [HMDB00195](http://www.hmdb.ca/metabolites/HMDB00195) | 6021 | 0.95 | 1.02 | 0.93 |
|  | hypoxanthine | LC/MS polar | 3127 | [C00262](http://www.genome.jp/dbget-bin/www_bget?cpd%2BC00262) | [HMDB00157](http://www.hmdb.ca/metabolites/HMDB00157) | 790 | **0.54** | 1.17 | **0.46** |
|  | xanthine | LC/MS polar | 3147 | [C00385](http://www.genome.jp/dbget-bin/www_bget?cpd%2BC00385) | [HMDB00292](http://www.hmdb.ca/metabolites/HMDB00292) | 1188 | 0.98 | **1.55** | **0.63** |
|  | xanthosine 5'-monophosphate (xmp) | LC/MS neg | 12024 | [C00655](http://www.genome.jp/dbget-bin/www_bget?cpd%2BC00655) | [HMDB01554](http://www.hmdb.ca/metabolites/HMDB01554) | 73323 | 0.75 | 1.00 | 0.75 |
|  | xanthosine | LC/MS neg | 15136 | [C01762](http://www.genome.jp/dbget-bin/www_bget?cpd%2BC01762) | [HMDB00299](http://www.hmdb.ca/metabolites/HMDB00299) | 64959 | 1.69 | **2.14** | 0.79 |
|  | N1-methylinosine | LC/MS pos early | 48351 |  | [HMDB02721](http://www.hmdb.ca/metabolites/HMDB02721) | 65095 | **2.71** | **1.36** | **2.00** |
|  | 2'-deoxyinosine | LC/MS neg | 15076 | [C05512](http://www.genome.jp/dbget-bin/www_bget?cpd%2BC05512) | [HMDB00071](http://www.hmdb.ca/metabolites/HMDB00071) | 65058 | **2.82** | **2.51** | 1.12 |
|  | urate | LC/MS neg | 1604 | [C00366](http://www.genome.jp/dbget-bin/www_bget?cpd%2BC00366) | [HMDB00289](http://www.hmdb.ca/metabolites/HMDB00289) | 1175 | **0.73** | 0.98 | **0.74** |
|  | uric acid ribonucleoside* | LC/MS neg | 62102 |  |  | 164933 | 1.08 | 1.56 | **0.70** |
|  | allantoin | LC/MS polar | 1107 | [C02350](http://www.genome.jp/dbget-bin/www_bget?cpd%2BC02350) | [HMDB00462](http://www.hmdb.ca/metabolites/HMDB00462) | 204 | **2.88** | 0.93 | **3.09** |

| **Sub Pathway** | **Biochemical Name** | **Platform** | **Comp ID** | **KEGG** | **HMDB** | **PubChem** | **Gal4>UAS**  **GAL4 Ctrl** | **Gal4>UAS**  **UAS Ctrl** | **UAS Ctrl GAL4 Ctrl** |
| --- | --- | --- | --- | --- | --- | --- | --- | --- | --- |
| Purine Metabolism, Adenine containing | adenosine 5'-diphosphate (ADP) | LC/MS neg | 3108 | [C00008](http://www.genome.jp/dbget-bin/www_bget?cpd%2BC00008) | [HMDB01341](http://www.hmdb.ca/metabolites/HMDB01341) | 6022 | 1.08 | **0.56** | **1.93** |
|  | adenosine 5'-monophosphate (AMP) | LC/MS pos early | 32342 | [C00020](http://www.genome.jp/dbget-bin/www_bget?cpd%2BC00020) | [HMDB00045](http://www.hmdb.ca/metabolites/HMDB00045) | 6083 | 1.13 | **0.65** | **1.74** |
|  | adenosine 3'-monophosphate (3'-AMP) | LC/MS neg | 35142 | [C01367](http://www.genome.jp/dbget-bin/www_bget?cpd%2BC01367) | [HMDB03540](http://www.hmdb.ca/metabolites/HMDB03540) | 41211 | 0.76 | 0.75 | 1.00 |
|  | adenosine-2',3'-cyclic monophosphate | LC/MS neg | 37467 | [C02353](http://www.genome.jp/dbget-bin/www_bget?cpd%2BC02353) | [HMDB11616](http://www.hmdb.ca/metabolites/HMDB11616) | 2024 | 1.01 | 0.87 | 1.17 |
|  | adenosine | LC/MS pos early | 555 | [C00212](http://www.genome.jp/dbget-bin/www_bget?cpd%2BC00212) | [HMDB00050](http://www.hmdb.ca/metabolites/HMDB00050) | 60961 | 1.00 | 0.98 | 1.02 |
|  | adenine | LC/MS pos early | 554 | [C00147](http://www.genome.jp/dbget-bin/www_bget?cpd%2BC00147) | [HMDB00034](http://www.hmdb.ca/metabolites/HMDB00034) | 190 | **0.59** | **0.85** | **0.70** |
|  | 1-methyladenine | LC/MS pos early | 1527 | [C02216](http://www.genome.jp/dbget-bin/www_bget?cpd%2BC02216) | [HMDB11599](http://www.hmdb.ca/metabolites/HMDB11599) | 78821 | **0.46** | 1.11 | **0.41** |
|  | N1-methyladenosine | LC/MS pos early | 15650 | [C02494](http://www.genome.jp/dbget-bin/www_bget?cpd%2BC02494) | [HMDB03331](http://www.hmdb.ca/metabolites/HMDB03331) | 27476 | 0.87 | 1.03 | **0.85** |
|  | N6-succinyladenosine | LC/MS pos early | 48130 |  | [HMDB00912](http://www.hmdb.ca/metabolites/HMDB00912) | 165243 | 0.96 | 0.72 | 1.32 |
| Purine Metabolism, Guanine containing | guanosine 5'- diphosphate (GDP) | LC/MS neg | 2848 | [C00035](http://www.genome.jp/dbget-bin/www_bget?cpd%2BC00035) | [HMDB01201](http://www.hmdb.ca/metabolites/HMDB01201) | 8977 | 0.80 | 0.69 | 1.16 |
|  | guanosine 5'- monophosphate (5'-GMP) | LC/MS neg | 2849 | [C00144](http://www.genome.jp/dbget-bin/www_bget?cpd%2BC00144) | [HMDB01397](http://www.hmdb.ca/metabolites/HMDB01397) | 6804 | 0.98 | **0.75** | **1.31** |
|  | guanosine-2',3'-cyclic monophosphate | LC/MS neg | 37139 | [C06194](http://www.genome.jp/dbget-bin/www_bget?cpd%2BC06194) | [HMDB11629](http://www.hmdb.ca/metabolites/HMDB11629) | 92823 | 1.01 | 0.75 | 1.35 |
|  | guanosine | LC/MS neg | 1573 | [C00387](http://www.genome.jp/dbget-bin/www_bget?cpd%2BC00387) | [HMDB00133](http://www.hmdb.ca/metabolites/HMDB00133) | 6802 | **1.26** | 1.18 | 1.07 |
|  | guanine | LC/MS pos early | 32352 | [C00242](http://www.genome.jp/dbget-bin/www_bget?cpd%2BC00242) | [HMDB00132](http://www.hmdb.ca/metabolites/HMDB00132) | 764 | 1.05 | 0.99 | 1.06 |
|  | 7-methylguanine | LC/MS pos early | 35114 | [C02242](http://www.genome.jp/dbget-bin/www_bget?cpd%2BC02242) | [HMDB00897](http://www.hmdb.ca/metabolites/HMDB00897) | 11361 | 0.99 | **1.20** | **0.82** |
|  | 2'-O-methylguanosine | LC/MS neg | 36811 | [C04545](http://www.genome.jp/dbget-bin/www_bget?cpd%2BC04545) |  |  | **1.53** | 1.18 | 1.30 |
|  | 7-methylguanosine | LC/MS pos early | 31580 | [C20674](http://www.genome.jp/dbget-bin/www_bget?cpd%2BC20674) |  |  | 1.15 | 0.91 | 1.26 |
|  | N2,N2-dimethylguanosine | LC/MS neg | 35137 |  | [HMDB04824](http://www.hmdb.ca/metabolites/HMDB04824) | 92919 | **1.86** | 1.11 | **1.68** |
|  | 2'-deoxyguanosine | LC/MS neg | 1411 | [C00330](http://www.genome.jp/dbget-bin/www_bget?cpd%2BC00330) | [HMDB00085](http://www.hmdb.ca/metabolites/HMDB00085) | 187790 | **1.36** | 1.22 | 1.12 |
| Pyrimidine Metabolism, Orotate containing | dihydroorotate | LC/MS polar | 601 | [C00337](http://www.genome.jp/dbget-bin/www_bget?cpd%2BC00337) | [HMDB03349](http://www.hmdb.ca/metabolites/HMDB03349) | 648 | **4.52** | **0.68** | **6.61** |
|  | orotate | LC/MS polar | 1505 | [C00295](http://www.genome.jp/dbget-bin/www_bget?cpd%2BC00295) | [HMDB00226](http://www.hmdb.ca/metabolites/HMDB00226) | 967 | **1.34** | 0.92 | **1.45** |
| Pyrimidine Metabolism, Uracil containing | uridine-2',3'-cyclic monophosphate | LC/MS neg | 37137 | [C02355](http://www.genome.jp/dbget-bin/www_bget?cpd%2BC02355) | [HMDB11640](http://www.hmdb.ca/metabolites/HMDB11640) | 439715 | 0.78 | 0.79 | 0.98 |
|  | uridine | LC/MS neg | 606 | [C00299](http://www.genome.jp/dbget-bin/www_bget?cpd%2BC00299) | [HMDB00296](http://www.hmdb.ca/metabolites/HMDB00296) | 6029 | **1.50** | **1.53** | 0.98 |
|  | uracil | LC/MS polar | 605 | [C00106](http://www.genome.jp/dbget-bin/www_bget?cpd%2BC00106) | [HMDB00300](http://www.hmdb.ca/metabolites/HMDB00300) | 1174 | 0.90 | 0.91 | 1.00 |
|  | pseudouridine | LC/MS neg | 33442 | [C02067](http://www.genome.jp/dbget-bin/www_bget?cpd%2BC02067) | [HMDB00767](http://www.hmdb.ca/metabolites/HMDB00767) | 15047 | **1.49** | **1.74** | 0.85 |
|  | 2'-O-methyluridine | LC/MS neg | 57655 |  |  | 102212 | **1.69** | **2.76** | **0.61** |
|  | 3-ureidopropionate | LC/MS pos early | 3155 | [C02642](http://www.genome.jp/dbget-bin/www_bget?cpd%2BC02642) | [HMDB00026](http://www.hmdb.ca/metabolites/HMDB00026) | 111 | 1.00 | **1.29** | 0.77 |
|  | beta-alanine | LC/MS pos early | 55 | [C00099](http://www.genome.jp/dbget-bin/www_bget?cpd%2BC00099) | [HMDB00056](http://www.hmdb.ca/metabolites/HMDB00056) | 239 | 1.21 | 1.00 | **1.21** |
|  | N-acetyl-beta-alanine | LC/MS polar | 37432 | [C01073](http://www.genome.jp/dbget-bin/www_bget?cpd%2BC01073) |  | 76406 | 1.07 | 1.20 | 0.90 |
| Pyrimidine Metabolism, Cytidine containing | cytidine 5'-monophosphate (5'-CMP) | LC/MS pos early | 2372 | [C00055](http://www.genome.jp/dbget-bin/www_bget?cpd%2BC00055) | [HMDB00095](http://www.hmdb.ca/metabolites/HMDB00095) | 6131 | 1.55 | 1.00 | **1.55** |
|  | cytidine 2' or 3'-monophosphate (2' or 3'-CMP) | LC/MS pos early | 61705 |  |  |  | 0.96 | 0.93 | 1.03 |
|  | cytidine 2',3'-cyclic monophosphate | LC/MS neg | 37465 | [C02354](http://www.genome.jp/dbget-bin/www_bget?cpd%2BC02354) | [HMDB11691](http://www.hmdb.ca/metabolites/HMDB11691) | 417654 | **0.76** | **0.82** | 0.92 |
|  | cytidine | LC/MS neg | 514 | [C00475](http://www.genome.jp/dbget-bin/www_bget?cpd%2BC00475) | [HMDB00089](http://www.hmdb.ca/metabolites/HMDB00089) | 6175 | **1.82** | **1.47** | 1.24 |
|  | cytosine | LC/MS pos early | 573 | [C00380](http://www.genome.jp/dbget-bin/www_bget?cpd%2BC00380) | [HMDB00630](http://www.hmdb.ca/metabolites/HMDB00630) | 597 | 1.05 | 1.39 | 0.76 |
|  | 3-methylcytidine | LC/MS pos early | 35132 |  |  | 159649 | **1.28** | **1.21** | 1.06 |
|  | 5-methylcytidine | LC/MS pos early | 22119 |  | [HMDB00982](http://www.hmdb.ca/metabolites/HMDB00982) | 92918 | **2.98** | 1.09 | **2.72** |
|  | 2'-O-methylcytidine | LC/MS pos early | 57554 |  |  | 150971 | **2.78** | **1.94** | **1.43** |
| Pyrimidine Metabolism, Thymine containing | 3-aminoisobutyrate | LC/MS pos early | 1566 | [C05145](http://www.genome.jp/dbget-bin/www_bget?cpd%2BC05145) | [HMDB03911](http://www.hmdb.ca/metabolites/HMDB03911) | 64956 | **0.56** | 0.96 | 0.59 |
| Purine and Pyrimidine Metabolism | methylphosphate | LC/MS pos early | 37070 |  | [HMDB61711](http://www.hmdb.ca/metabolites/HMDB61711) | 13130 | 1.17 | 0.94 | 1.24 |
| Dinucleotide | (3'-5')-adenylyluridine | LC/MS neg | 52740 |  |  | 112074 | 1.00 | 1.00 | 1.00 |
| Nicotinate and Nicotinamide Metabolism | nicotinate | LC/MS pos early | 1504 | [C00253](http://www.genome.jp/dbget-bin/www_bget?cpd%2BC00253) | [HMDB01488](http://www.hmdb.ca/metabolites/HMDB01488) | 938 | 0.93 | **1.21** | **0.77** |
|  | nicotinamide ribonucleotide (NMN) | LC/MS pos early | 22152 | [C00455](http://www.genome.jp/dbget-bin/www_bget?cpd%2BC00455) | [HMDB00229](http://www.hmdb.ca/metabolites/HMDB00229) | 14180 | 1.11 | 1.05 | 1.06 |
|  | nicotinamide riboside | LC/MS pos early | 33013 | [C03150](http://www.genome.jp/dbget-bin/www_bget?cpd%2BC03150) | [HMDB00855](http://www.hmdb.ca/metabolites/HMDB00855) | 439924 | 1.37 | 2.01 | 0.68 |
|  | nicotinamide adenine dinucleotide (NAD+) | LC/MS pos early | 5278 | [C00003](http://www.genome.jp/dbget-bin/www_bget?cpd%2BC00003) | [HMDB00902](http://www.hmdb.ca/metabolites/HMDB00902) | 5893 | 0.90 | **0.76** | **1.19** |
|  | nicotinate adenine dinucleotide (NAAD+) | LC/MS neg | 15725 |  |  | 25246170 | 0.88 | 0.75 | 1.18 |
|  | trigonelline (N'-methylnicotinate) | LC/MS pos early | 32401 | [C01004](http://www.genome.jp/dbget-bin/www_bget?cpd%2BC01004) | [HMDB00875](http://www.hmdb.ca/metabolites/HMDB00875) | 5570 | 1.11 | 0.90 | 1.23 |
| Riboflavin Metabolism | riboflavin (Vitamin B2) | LC/MS pos early | 1827 | [C00255](http://www.genome.jp/dbget-bin/www_bget?cpd%2BC00255) | [HMDB00244](http://www.hmdb.ca/metabolites/HMDB00244) | 493570 | **2.72** | **0.71** | **3.84** |
|  | flavin adenine dinucleotide (FAD) | LC/MS neg | 2134 | [C00016](http://www.genome.jp/dbget-bin/www_bget?cpd%2BC00016) | [HMDB01248](http://www.hmdb.ca/metabolites/HMDB01248) | 643975 | **0.73** | 0.74 | 0.99 |
| Pantothenate and CoA Metabolism | pantothenate | LC/MS neg | 1508 | [C00864](http://www.genome.jp/dbget-bin/www_bget?cpd%2BC00864) | [HMDB00210](http://www.hmdb.ca/metabolites/HMDB00210) | 6613 | 0.75 | 0.91 | 0.82 |
|  | pantetheine | LC/MS pos early | 57555 | [C00831](http://www.genome.jp/dbget-bin/www_bget?cpd%2BC00831) |  | 439322 | **0.52** | **0.75** | **0.70** |
| Ascorbate and Aldarate Metabolism | ascorbate (Vitamin C) | LC/MS pos early | 32354 | [C00072](http://www.genome.jp/dbget-bin/www_bget?cpd%2BC00072) | [HMDB00044](http://www.hmdb.ca/metabolites/HMDB00044) |  | **1.63** | **1.24** | **1.31** |
|  | dehydroascorbate | LC/MS polar | 1659 | [C05422](http://www.genome.jp/dbget-bin/www_bget?cpd%2BC05422) | [HMDB01264](http://www.hmdb.ca/metabolites/HMDB01264) | 835 | 0.58 | 0.70 | 0.82 |
|  | threonate | LC/MS polar | 27738 | [C01620](http://www.genome.jp/dbget-bin/www_bget?cpd%2BC01620) | [HMDB00943](http://www.hmdb.ca/metabolites/HMDB00943) | 151152 | 0.97 | 1.11 | 0.87 |
|  | gulonate* | LC/MS polar | 46957 | [C00257](http://www.genome.jp/dbget-bin/www_bget?cpd%2BC00257) | [HMDB03290](http://www.hmdb.ca/metabolites/HMDB03290) | 9794176 | **0.75** | 1.01 | **0.74** |
| Tocopherol Metabolism | alpha-tocopherol | LC/MS pos late | 1561 | [C02477](http://www.genome.jp/dbget-bin/www_bget?cpd%2BC02477) | [HMDB01893](http://www.hmdb.ca/metabolites/HMDB01893) | 14985 | 1.09 | **1.23** | 0.89 |
|  | gamma-tocopherol/beta-tocopherol | LC/MS pos late | 52473 |  |  |  | **1.58** | **1.51** | 1.04 |
| Biotin Metabolism | biotin | LC/MS pos early | 568 | [C00120](http://www.genome.jp/dbget-bin/www_bget?cpd%2BC00120) | [HMDB00030](http://www.hmdb.ca/metabolites/HMDB00030) | 171548 | 1.31 | 1.20 | 1.09 |
| Tetrahydrobiopterin Metabolism | biopterin | LC/MS neg | 12358 | [C06313](http://www.genome.jp/dbget-bin/www_bget?cpd%2BC06313) | [HMDB00468](http://www.hmdb.ca/metabolites/HMDB00468) | 445040 | **0.67** | 0.85 | **0.79** |
|  | dihydrobiopterin | LC/MS pos early | 35129 | [C00268](http://www.genome.jp/dbget-bin/www_bget?cpd%2BC00268) | [HMDB00038](http://www.hmdb.ca/metabolites/HMDB00038) | 1879 | **0.52** | **0.78** | **0.66** |
| Pterin Metabolism | isoxanthopterin | LC/MS pos early | 27732 | [C03975](http://www.genome.jp/dbget-bin/www_bget?cpd%2BC03975) | [HMDB00704](http://www.hmdb.ca/metabolites/HMDB00704) | 10729 | 0.82 | 0.76 | 1.09 |
|  | pterin | LC/MS neg | 43023 | [C00715](http://www.genome.jp/dbget-bin/www_bget?cpd%2BC00715) | [HMDB00802](http://www.hmdb.ca/metabolites/HMDB00802) | 73000 | **1.23** | 1.12 | 1.09 |
|  | sepiapterin | LC/MS pos early | 48139 | [C00835](http://www.genome.jp/dbget-bin/www_bget?cpd%2BC00835) | [HMDB00238](http://www.hmdb.ca/metabolites/HMDB00238) | 65253 | **0.54** | **0.83** | **0.65** |
|  | xanthopterin | LC/MS polar | 54728 |  |  | 8397 | 0.88 | **0.70** | **1.26** |
| Hemoglobin and Porphyrin Metabolism | 5-aminolevulinate | LC/MS pos early | 2290 | [C00430](http://www.genome.jp/dbget-bin/www_bget?cpd%2BC00430) | [HMDB01149](http://www.hmdb.ca/metabolites/HMDB01149) | 137 | 0.72 | 1.67 | **0.43** |
| Thiamine Metabolism | thiamin (Vitamin B1) | LC/MS pos early | 5341 | [C00378](http://www.genome.jp/dbget-bin/www_bget?cpd%2BC00378) | [HMDB00235](http://www.hmdb.ca/metabolites/HMDB00235) | 1130 | **0.61** | **0.56** | 1.09 |
|  | thiamin monophosphate | LC/MS pos early | 15798 | [C01081](http://www.genome.jp/dbget-bin/www_bget?cpd%2BC01081) | [HMDB02666](http://www.hmdb.ca/metabolites/HMDB02666) | 3382778 | 0.92 | **0.61** | **1.51** |
| Vitamin A Metabolism | carotene diol (1) | LC/MS pos late | 57635 |  |  |  | 0.88 | 1.10 | 0.80 |
|  | carotene diol (2) | LC/MS pos late | 57636 |  |  |  | 0.78 | 1.25 | **0.62** |
|  | carotene diol (3) | LC/MS pos late | 57637 |  |  |  | **0.52** | 1.09 | **0.48** |
| Vitamin B6 Metabolism | pyridoxal | LC/MS pos early | 1651 | [C00250](http://www.genome.jp/dbget-bin/www_bget?cpd%2BC00250) | [HMDB01545](http://www.hmdb.ca/metabolites/HMDB01545) | 1050 | 1.10 | 0.94 | 1.16 |
|  | pyridoxate | LC/MS neg | 31555 | [C00847](http://www.genome.jp/dbget-bin/www_bget?cpd%2BC00847) | [HMDB00017](http://www.hmdb.ca/metabolites/HMDB00017) | 6723 | 1.48 | 0.96 | **1.55** |
| Benzoate Metabolism | 4-hydroxyhippurate | LC/MS neg | 35527 |  | [HMDB13678](http://www.hmdb.ca/metabolites/HMDB13678) | 151012 | **0.34** | 1.36 | **0.25** |
|  | 4-hydroxybenzoate | LC/MS neg | 21133 | [C00156](http://www.genome.jp/dbget-bin/www_bget?cpd%2BC00156) | [HMDB00500](http://www.hmdb.ca/metabolites/HMDB00500) | 135 | **0.69** | 1.06 | **0.65** |
| Food Component/Plant | 2,3-dihydroxyisovalerate | LC/MS polar | 38276 | [C04039](http://www.genome.jp/dbget-bin/www_bget?cpd%2BC04039) | [HMDB12141](http://www.hmdb.ca/metabolites/HMDB12141) | 677 | **0.39** | 1.13 | **0.35** |
|  | 2-isopropylmalate | LC/MS polar | 15667 | [C02504](http://www.genome.jp/dbget-bin/www_bget?cpd%2BC02504) | [HMDB00402](http://www.hmdb.ca/metabolites/HMDB00402) | 77 | 0.87 | 0.84 | 1.04 |
|  | gluconate | LC/MS polar | 587 | [C00257](http://www.genome.jp/dbget-bin/www_bget?cpd%2BC00257) | [HMDB00625](http://www.hmdb.ca/metabolites/HMDB00625) | 10690 | 0.88 | 0.87 | 1.01 |
|  | ergothioneine | LC/MS pos early | 37459 | [C05570](http://www.genome.jp/dbget-bin/www_bget?cpd%2BC05570) | [HMDB03045](http://www.hmdb.ca/metabolites/HMDB03045) | 3032311 | 1.10 | 1.17 | 0.94 |
|  | erythritol | LC/MS polar | 20699 | [C00503](http://www.genome.jp/dbget-bin/www_bget?cpd%2BC00503) | [HMDB02994](http://www.hmdb.ca/metabolites/HMDB02994) | 222285 | **0.76** | 1.03 | **0.74** |
|  | kojibiose | LC/MS polar | 21040 | [C19632](http://www.genome.jp/dbget-bin/www_bget?cpd%2BC19632) | [HMDB11742](http://www.hmdb.ca/metabolites/HMDB11742) | 164939 | 1.20 | 1.14 | 1.06 |
|  | panose | LC/MS polar | 37284 | [C00713](http://www.genome.jp/dbget-bin/www_bget?cpd%2BC00713) | [HMDB11729](http://www.hmdb.ca/metabolites/HMDB11729) | 5288421 | **1.23** | **0.87** | **1.42** |
|  | quinate | LC/MS polar | 18335 | [C00296](http://www.genome.jp/dbget-bin/www_bget?cpd%2BC00296) | [HMDB03072](http://www.hmdb.ca/metabolites/HMDB03072) | 6508 | 0.80 | 1.03 | 0.78 |
|  | stachydrine | LC/MS pos early | 34384 | [C10172](http://www.genome.jp/dbget-bin/www_bget?cpd%2BC10172) | [HMDB04827](http://www.hmdb.ca/metabolites/HMDB04827) | 115244 | **0.52** | **0.55** | 0.95 |
|  | methyl glucopyranoside (alpha + beta) | LC/MS pos early | 46144 |  |  |  | 1.10 | 0.98 | 1.13 |
|  | 2-keto-3-deoxy-gluconate | LC/MS polar | 48141 | [C00204](http://www.genome.jp/dbget-bin/www_bget?cpd%2BC00204) | [HMDB01353](http://www.hmdb.ca/metabolites/HMDB01353) | 161227 | 0.68 | **0.43** | 1.58 |
| Drug - Topical Agents | salicylate | LC/MS polar | 1515 | [C00805](http://www.genome.jp/dbget-bin/www_bget?cpd%2BC00805) | [HMDB01895](http://www.hmdb.ca/metabolites/HMDB01895) | 338 | **0.59** | **0.67** | 0.88 |
| Chemical | succinimide | LC/MS polar | 41888 | [C07273](http://www.genome.jp/dbget-bin/www_bget?cpd%2BC07273) |  | 11439 | 1.26 | 1.11 | 1.14 |
|  | thioproline | LC/MS pos early | 53231 |  |  | 93176 | 0.77 | 1.00 | 0.77 |

**Table 1-supplement table 1:** **Metabolomics of *Repo*>20x*Shibire* fly heads.**

All measured metabolites and their respective categories are listed for samples from *Repo*-*GAL4*>UAS-20x*Shibire*, and both parental controls. Welch’s t-test was performed on scaled signal for each metabolite, comparing the conditions shown. Green highlighting marks a significant difference (p≤0.05) between the groups, where metabolite ratio is <1.00, while light green is not significant, but close to the threshold (0.05<p<0.10). Red highlighting marks a significant difference (p≤0.05) between groups where metabolite ratio is ≥ 1.00, and light red is not significant, but close to the threshold (0.05<p<0.10).
